# Marmosets as model systems for the study of Alzheimer's disease and related dementias: Substantiation of physiological tau 3R and 4R isoform expression and phosphorylation

**DOI:** 10.1002/alz.14366

**Published:** 2024-11-19

**Authors:** Hasi Huhe, Sarah M. Shapley, Duc M. Duong, Fang Wu, Seung‐Kwon Ha, Sang‐Ho Choi, Julia Kofler, Yongshan Mou, Thais Rafael Guimaraes, Amantha Thathiah, Caroline M. Watson, Lauren K. H. Schaeffer, Gregory W. Carter, Nicholas T. Seyfried, Afonso C. Silva, Stacey J. Sukoff Rizzo

**Affiliations:** ^1^ Aging Institute University of Pittsburgh School of Medicine Pittsburgh Pennsylvania USA; ^2^ Department of Biochemistry Emory University School of Medicine Atlanta Georgia USA; ^3^ Department of Neurobiology University of Pittsburgh School of Medicine Pittsburgh Pennsylvania USA; ^4^ Department of Pathology University of Pittsburgh School of Medicine Pittsburgh Pennsylvania USA; ^5^ The Jackson Laboratory Bar Harbor Maine USA

**Keywords:** Alzheimer's disease, biomarkers, marmosets, tau

## Abstract

**INTRODUCTION:**

Marmosets spontaneously develop pathological hallmarks of Alzheimer's disease (AD) including amyloid beta plaques. However, tau expression in the marmoset brain has been understudied.

**METHODS:**

Isoforms of tau were examined by western blot, mass spectrometry, immunofluorescence, and immunohistochemical staining.

**RESULTS:**

3R and 4R tau isoforms are expressed in marmoset brains at both the transcript and protein levels across ages. Mass spectrometry analysis revealed that tau peptides in marmoset corresponded to the 3R and 4R peptides in human brain, with 3R predominating at birth and an ≈40%:60% 3R:4R ratios in adolescents and adults; tau was distributed widely in neurons, with localization in the soma and synaptic regions. Phosphorylation residues were observed on Threonine (Thr) Thr181, Thr217, Thr231, Serine (Ser) Ser202/Thr205, and Ser396/Ser404.

**DISCUSSION:**

Our results confirm both 3R and 4R tau isoform expression and phosphorylation residues in the marmoset brain, and emphasize the significance of marmosets with natural expression of AD‐related hallmarks as important translational models for AD.

**Highlights**

- We report comprehensive characterization of tau isoform expression in marmoset brains across the lifespan.
- 3R and 4R tau isoforms are expressed in marmoset brains at both the transcript and protein levels across ages.
- These data emphasize the significance of marmosets with natural expression of primate‐specific traits that are important for the study of Alzheimer's disease.

## 1 INTRODUCTION

Non‐human primates provide critical insight into primate‐specific mechanisms that are the etiologies of human diseases. Given the translational limitations of rodent models, the common marmoset (*Callithrix jacchus*) has emerged as an important model system for studying diseases of aging, including Alzheimer's disease (AD).^1^, ^2^, ^3^, ^4^, ^5^ The marmoset brain shares a number of similarities with the human brain, including neuroanatomy and neurocircuitry,^6^, ^7^ as well as age‐related cognitive decline and neuropathological features in the marmoset that align with postmortem tissues of human AD patients.^1^, ^8^, ^9^ One of the major pathological hallmarks of AD, amyloid beta (Aβ) deposition in the brain, has also been reported frequently in marmosets as early as 7 years of age, which is equivalent to a 56‐year‐old human.^8^ Despite the well‐reported characterization of Aβ in the aging marmoset brain, only a few studies have comprehensively characterized tau in the marmoset brain.^9^, ^10^, ^11^, ^12^

A microtubule‐associated protein tau (MAPT) encoded by the *MAPT* gene, is expressed predominantly in the brain, and plays essential roles in neuronal function. During development, alternative splicing of *MAPT* results in six isoforms of tau transcripts in humans, which include 0N3R, 1N3R, 2N3R, 0N4R, 1N4R, and 2N4R. In humans, fetal and neonatal brains exclusively express 0N3R tau, whereas alternative splicing of exon 10 results in the expression of both 3R and 4R isoforms in the adult brain, which are maintained throughout the lifespan.^13^, ^14^, ^15^, ^16^ Under pathological conditions, including as a consequence of neurodegeneration, differential expression of the 3R and 4R tau isoforms is observed across different brain regions, and depending on the predominance of the isoform, may confer any one of several distinct tauopathies, including in AD.^17^, ^18^, ^19^, ^20^, ^21^ In humans, physiological tau is not only localized in axons but importantly also in dendrites.^22^ Recent data in macaques have elegantly demonstrated the progression of tau aggregation and subsequent fibrillation in the synaptic region, which may begin‐in dendrites and consequently leads to alterations in synaptic plasticity,^23^, ^24^, ^25^, ^26^, ^27^, ^29^, ^30^ including long‐term depression (LTD) formation, which is necessary for cognitive function.^31^, ^32^, ^33^, ^34^, ^35^, ^36^, ^37^ The formation of pathological tau aggregates, including in AD, is tightly associated with post‐translational modifications of tau, especially hyperphosphorylation.^38^, ^39^, ^40^ It is well established that tau hyperphosphorylation plays an essential role in facilitating pathological tau aggregation and that phosphorylation residues change during AD progression.^39^, ^40^ Related to this, accumulation of hyperphosphorylated and misfolded tau in synapses^26^, ^36^, ^39^ may contribute to impaired synaptic function.^33^, ^34^, ^35^, ^36^, ^37^ For example, Threonine (Thr) Thr 181, Thr 217, and Thr 231 hyperphosphorylation has been reported in the early preclinical stages of AD,^41^ whereas AT8 Serine (Ser) (Ser202/Thr205) and PHF1 (Ser396/Ser404) hyperphosphorylation are reported in later stages of AD.^42^, ^43^

Although several studies have characterized tau extensively in other non‐human primate species, including macaques as described above, and have exquisitely demonstrated alignment with human tau,^14^ this has not yet been studied comprehensively in marmosets. Of the few studies in marmosets that have reported tau pathology and phosphorylation sites,^9^, ^10^, ^11^ only one study investigated tau isoform expression in a limited number of subjects.^10^ Consequently, several critical gaps remain concerning tau expression in the marmoset and its relevance to AD: specifically, age‐dependent alterations of tau isoform expression and phosphorylation residues related to pathological tau aggregation, as well as the subcellular distribution of tau in the marmoset brain, which have yet to be comprehensively investigated. The present study aimed to investigate physiological 3R and 4R tau expression comprehensively in brain tissues of unrelated marmosets across different age groups, subcellular distribution of tau, as well as the aggregation‐related phosphorylation residues and their properties, which is critical foundational knowledge for understanding the relevance of marmosets as model systems for the study of AD.

## 2 METHODS

### 2.1 Subjects

All experimental procedures involving animals were performed in accordance with state and federal laws, locally approved by the University of Pittsburgh Institutional Animal Care and Use Committee (IACUC), and were in line with and strictly adhered to the Guide for the Care and Use of Laboratory Animals.^44^

#### 2.1.1 Marmosets

Outbred male and female common marmosets (*Callihrix jacchus*) were housed in an the Association for Assessment and Accreditation of Laboratory Animal Care International (AAALAC)‐accredited facility at the University of Pittsburgh. Subjects were socially housed, typically in pairs or family groups, and maintained at a temperature range of 76°F –78°F and 30%–70% humidity, with a 12 h:12 h light‐to‐dark cycle (lights on at 7 am). Subjects were fed a diet consisting of twice daily provisions of commercial chow, including a purified diet, and supplemented with fresh fruit and vegetables daily, with drinking water provided ad libitum. In addition, foraging materials and enrichment were provided daily. Subject demographics for the present studies are provided in Table 1, which includes subjects spanning an age range from post‐natal day (PND) 1 to 12 years.

**TABLE 1 alz14366-tbl-0001:** Demographics of marmoset tissues analyzed in the present studies.

Subject ID	Sex	Age at tissue collection	Analysis and figure
AP	Female	Neonate (Postnatal Day 1)	Western blot (Figure 1B&C) Mass spectrometry (Figure 2, S3, S4) RT‐PCR (Figure 1A), qPCR (Figure S2)
MO	Male	12 months	Western blot (Figure 1, 5) Mass spectrometry (Figure 2, S3, S4)
WI	Female	13 months	Western blot (Figure 1, 5) Mass spectrometry (Figure 2, S3, S4)
VI	Female	13 months	Western blot (Figure 1, 5) Mass spectrometry (Figure 2, S3, S4) RT‐PCR (Figure 1 A), qPCR (Figure S2)
HU	Male	7 years	Western blot (Figure 1, 4, 5A&B) Mass spectrometry (Figure 2, S3, S4)
AR	Female	7 years	Western blot (Figure 1, 4, 5A&B) Mass spectrometry (Figure 2, S3, S4)
ST	Female	9 years	Western blot (Figure 1, 4, 5A&B) Mass spectrometry (Figure 2, S3, S4) RT‐PCR (Figure 1 A), qPCR (Figure S2)
CO	male	12 years	Immunofluorescence staining (Figure 3, S5) Immunohistochemistry staining (Figure S6)

#### 2.1.2 Mice

Breeding pairs of C57BL/6J mice (JAX# 000664) were obtained from the Jackson Laboratory (Bar Harbor, ME) at 8–12 weeks of age. Mice were group housed (*n* = 2–4 per cage) with ad libitum food and water in a dedicated mouse housing room with a 12:12 light‐to‐dark cycle (lights on at 7 am). Male offspring (*N* = 3), ages 3 weeks, 13 months, and 17 months were used for these studies. All experiments were conducted during the light cycle.

### 2.2 Tissue preparation

Brain tissues from unrelated outbred male and female marmosets and inbred C57BL/6J mice were obtained following humane euthanasia in accordance with the American Veterinary Medical Association (AVMA) Guidelines for the euthanasia of animals (https://www.avma.org/resources‐tools/avma‐policies/avma‐guidelines‐euthanasia‐animals). Briefly, mice (*n* = 3) were anesthetized by isoflurane inhalant anesthesia (3%–5% in O_2_) to the surgical plane of anesthesia, and the brain was dissected and flash‐frozen following decapitation. Marmosets were sedated and anesthetized by intramuscular injection of ketamine (20–40 mg/kg) and intravenous injection of sodium pentobarbital (10–30 mg/ kg), respectively. Brain hemispheres were extracted following transcardial perfusion with ice‐cold phosphate‐buffered saline (PBS) for immunofluorescence staining and immunohistochemistry, then fixed in 10% neutral buffered formalin (NBF; Sigma Aldrich) until analysis. For biochemical analysis, brain hemispheres were extracted rapidly after perfusion, snap frozen by isopentane with dry ice, and stored at −80°C until analysis. Tissue was collected within 12 h of parturition from *n *= 1 neonate found dead and immediately frozen and stored at −80°C until analysis. Given the long postmortem interval for the neonate, these data were excluded in phosphorylation site analyses. For protein extraction, the entorhinal cortex, hippocampus, and prefrontal cortex (PFC) were isolated from frozen sagittal right hemisphere sections, according to the marmoset brain atlas (2dviewer.marmosetbrainmapping.org/Atlas_vP_show.html). Briefly, PFC was obtained by coronally sectioning 3−3.5  mm brain tissue from the frontal cortex, which contains dorsolateral prefrontal cortex, ventrolateral prefrontal cortex, medial prefrontal cortex, orbital frontal cortex, and anterior prefrontal cortex. Entire entorhinal cortex (≈1.5 × 7 × 3 mm3) was dissected from right hemisphere and used for biochemical analysis.

**RESEARCH IN CONTEXT**

1. **Systematic review**: Using traditional sources (e.g., PubMed, conference abstracts) the authors searched for studies characterizing tau isoform expression in marmosets.
2. **Interpretation**: The marmoset has emerged as an important model for the study of neurodegenerative disorders including Alzheimer's disease (AD). Despite a wealth of data characterizing amyloid in the brains of marmosets, tau has been understudied. We demonstrate the expression of both 3R and 4R tau isoforms in the brains of marmosets across a span of age ranges at both the transcript and protein levels, with 3R predominating at birth and an ≈40%:60% 3R:4R ratio in adolescence and adulthood.
3. **Future directions**: Marmosets are important model systems for studying primate‐specific mechanisms that underlie neurodegenerative diseases, and have utility for evaluating interventions aimed at stopping and preventing disease.

Frozen inferior temporal cortex tissue from a de‐identified AD patient donor with confirmed tau pathology was obtained from the Department of Pathology at the University of Pittsburgh, following Committee for Oversight of Research and Clinical Training Involving Decedents (CORID) approval and used as control tissue for western blot analysis. Postmortem frozen human brain of AD (*n* = 4) and non‐demented control sections (*n* = 4) of the dorsolateral prefrontal cortex were obtained from the Emory Alzheimer's Disease Research Center brain bank (pathological traits described in Table S1A) and used for mass spectrometry and western blot as indicated in Table S1B.[Table alz14366-tbl-0001]

### 2.3 RNA isolation and reverse transcription cDNA

Prefrontal cortex tissue from *n* = 3 marmosets (50−70 mg), including a neonate, an adolescent (13 months), and an aged adult (9 years), were used for RNA extraction. Frozen brain sections were thawed on the wet ice, and total RNA was extracted by TRIzol Plus RNA purification kit (Invitrogen #12183555) according to the manufacturer's protocol. On‐column DNase treatment (PureLink DNase Set, ThermoFisher Scientific #12185010) was performed to obtain DNA‐free total RNA. The yields of the total RNA for each sample were determined using a Nanodrop one spectrophotometer (Thermo Fisher Scientific). One hundred nanograms of total RNA and oligo(dT)20 primer were used for reverse transcription by SuperScript III First‐Strand Synthesis System (Thermo Fisher Scientific #18080‐051) according to the manufacturer‐provided protocol to construct the complementary DNA (cDNA) library.

### 2.4 RT‐PCR analysis and *MAPT* sequencing

A reverse‐transcribed cDNA library was used as a template to amplify *MAPT* isoforms (with and without exon10) using reverse transcriptase polymerase chain reaction (RT‐PCR) Basic Local Alignment Search Tool (BLAST). The reaction was carried out by DreamTaq Hot Start Green DNA Polymerase (Thermo Fisher Scientific #MAN0015979). The PCR reaction was executed in ThermalCycler9 (Bio‐Rad) under a touchdown PCR program (annealing temperature from 68°C to 63°C decrements of 0.5°C in every cycle for 10 cycles, followed by 63°C for 25 cycles). The primer used in the reaction was designed based on marmoset 2N4R tau mRNA sequence (NCBI database (MK630008): forward primer GTCAAGTCCAAGATCGGTTC; reverse primer TGGTCTGTCTTGGCTTTGGC. The PCR products were analyzed by electrophoresis on 4% (w/v) agarose gels. The amplified DNA products were labeled with GelGreen Nucleic Acid Gel Stain dye (Biotium #41004) and visualized by ChemiDoc Imaging Systems (Bio‐Rad, California, USA). Following visualization, each *MAPT* DNA band was excised from agarose gel, and DNA was purified by gel extraction kit (Qiagen #2874 Maryland, USA) according to the manufacturer's instructions. The purified DNA fragments were sequenced by Genewhiz (AZENTA life sciences, MA, USA) from both the 5′ and 3′ terminals. The *MAPT* isoforms were confirmed using the Nucleotide BLAST program (National Institutes of Health [NIH], National Library of Medicine).

### 2.5 Extraction of Sarkosyl soluble and insoluble tau

The Sarkosyl soluble and insoluble fractions were extracted from the hippocampus, entorhinal cortex, and PFC of marmoset frozen brain tissues and the frozen human AD brain cortex, similar to methods described previously.^45^ Briefly, the brain tissue was homogenized using a Potter‐Elvehjem tissue homogenizer in nine volumes (wt/vol) of Tris homogenized buffer: 10  mM Tris‐HCl, pH 7.4, 0.8 M  NaCl, 10% sucrose, 1  mM EGTA (ethylene glycol tetraacetic acid), 2  mM DTT (dithiothreitol), Ethylenediaminetetraaceticacid (EDTA‐free Pierce Protease Inhibitor Mini Tablet (Thermo Fisher Scientific #A32955), 1x phosphatase inhibitor cocktail I (Abcam, Waltham, MA #ab201112) with 0.1% Sarkosyl added, and centrifuged at 10,000 × *g* for 10 min at 4°C. Pellets were re‐extracted using half the volume of the homogenization buffer, and the resulting supernatants were pooled (S1). Additional Sarkosyl was added to the supernatant (S1) to reach a final concentration of 1% and rotated for an additional 1 h at 4°C, followed by 60 min of centrifugation at 300,000 × *g* at 4°C. The resulting pellets were resuspended in 100 µL PBS as Sarkosyl‐insoluble fraction (P2). The supernatants are Sarkosyl‐soluble fraction (S2). Each fraction (S2, P2) was analyzed by western blotting.

### 2.6 Western blotting (WB)

For western blotting, the protein concentration of various fractions from different subjects was determined using a bicinchoninic acid (BCA) assay (Thermo Fisher Scientific). Proteins were denatured by heating at 95°C for 5 min, adding 1x Laemmli sample buffer with 2.5% of 2‐mercaptoethanol. Equivalent amounts of protein were loaded on 4%–15% or 4%–20% Mini‐PROTEAN precast TGX gel (Bio‐Rad). After electrophoresis, proteins were transferred to the nitrocellulose membrane using a turbo transfer system (Bio‐Rad). The membranes were further blocked with EveryBlot blocking buffer (Bio‐Rad #12010020) for 30  min at room temperature and incubated with primary antibodies overnight at 4°C. After washes (3x for 10 min), the membranes were incubated for 1 h in fluorescent‐dye conjugated or HRP (horseradish peroxidase) conjugated anti‐rabbit or anti‐mouse secondary antibodies. The HRP‐labeled membrane was further incubated with Pierce ECL substrate (Thermo Fisher Scientific #32106) or SuperSignal West Pico PLUS Chemiluminescent Substrate (Thermo Fisher Scientific #34579). The target protein expression was visualized by ChemiDoc MP Imaging System (Bio‐Rad). GAPDH was used as a loading control. Primary antibodies used for western blotting in this study were: mouse anti‐4R tau monoclonal antibody‐RD4 (1:800; Millipore Sigma #05‐804); mouse anti‐3R tau monoclonal antibody‐RD3 (1:800; Millipore Sigma #05‐803); rabbit anti‐4R tau monoclonal antibody (1:1000; Cell Signaling #79327); mouse anti‐tau monoclonal antibody‐Tau5 (1:500; Thermo Fisher #AHB0042); mouse anti‐human tau monoclonal antibody‐HT7 (1:2000; Thermo Fisher #MN1000); mouse anti‐Ser396/Ser404 pTau monoclonal antibody‐PHF1 (1:1000; gifted from the laboratory of Dr Peter Davies, Department of Pathology, Albert Einstein College of Medicine, NY, USA); mouse anti‐pTau (Ser202/Thr205) Monoclonal Antibody‐AT8 (1:500; Thermo Fisher #MN1020); mouse anti‐pTau (Thr231) monoclonal antibody‐AT180 (1:500; Thermo Fisher #MN1040); mouse anti‐tau oligomeric antibody‐TOMA1 (1:300; Thermo Fisher #MABN819); mouse anti‐pTau (Thr181) monoclonal antibody‐AT270 (1:1000; Thermo Fisher #MN1050); rabbit anti–pTau (Thr217) monoclonal antibody (1:1000; Cell Signaling #51625); hFAB rhodamine conjugated anti‐GAPDH antibody (1:2000; Bio‐Rad #12004168); mouse anti‐PSD95 monoclonal antibody (1:1000; Abcam #ab2723); and mouse anti‐synaptophysin monoclonal antibody (1:2000; ThermoFisher Scientific #MA1‐213). Secondary antibodies used in this study were: HRP‐conjugated goat‐anti‐mouse immunoglobulin G (IgG) secondary antibody (1:10000; Jackson ImmunoResearch #115‐035‐003) or Donkey Anti‐Rabbit IgG (1:10000; Jackson ImmunoResearch #711‐035‐152). For AD positive control, 0.5 µg of insoluble fraction was loaded, whereas for all other marmoset samples, an equivalent 15 µg total insoluble protein was loaded in each well.

### 2.7 Mass spectrometry (MS)

Marmoset (*n* = 7), mouse (*n* = 1), and human (*n* = 8) brain sections were analyzed by mass spectrometry (MS) and illustrated in Figure S1. The PFC (Brodmann's Area 9) of frozen brain sections from human AD patients and non‐demented controls were obtained from the Emory Alzheimer's Disease Research Center brain bank (pathological traits described in Table S1). Marmoset, mouse, and human brain tissues were homogenized as described.^46^, ^47^ Briefly, human brain, marmoset (PFC), and mouse hemi brain were homogenized in urea homogenization buffer (10 mM Tris, 100 mM NaH2PO4, 8 M Urea, pH 8.5 with 1x Halt protease inhibitor (Thermo Fisher) with ≈100 µL stainless‐steel beads (0.9 to 2.0 mm NextAdvance) by a bullet blender at 4°C for two full 5‐min intervals. Lysates were transferred to fresh tubes and sonicated three times at 30% amplitude on ice for 15 s with 5 s intervals. Lysates were centrifuged at 4°C for 5 min at 15,000 × *g*. Supernatants were collected and used for MS. The protein concentrations were determined through BCA (Pierce). The lysates were further reduced with 5 mM DTT and alkylated with 10 mM iodoacetamide (IAA) at room temperature for 30  min, respectively, followed by diluting urea concentration to < 1 M by adding 100 mM Tris‐HCl, pH 8.0 buffer (v/v = 9:1), and 1 mM CaCl_2_ buffer. Briefly, 60 µg of total protein per sample and 5 µg recombinant tau (rTau) (2N4R tau and 1N3R tau) were digested with either Trypsin Protease (Pierce) or LysargiNase (EMD Millipore) at 1:50 w/w overnight at room temperature.

The resulting peptides were acidified 1:9 (volume per volume, v/v) with acidification buffer (10% formic acid [FA] and 1% trifluoroacetic acid [TFA]) to quench enzyme activity. Custom PEPoTec heavy peptides of PRM targets containing terminal heavy lysine, K (+8 Da), or arginine, R (+10 Da) were purchased from Thermo Fisher. Relative peptide signals for pooling were determined by serial dilutions in Serial Reaction Monitoring (SRM) mode on an Agilent Triple Quadrupole 6495C and calculated to be within an order of intensity to biological (light) signal. Pre‐optimized amounts of peptide pools were added to acidified peptides and desalted by loading the peptides onto a 10 mg Oasis PRiME Hydrophilic‐Lipiphilic Balance (HLB) 96‐well plate (Waters), followed by washing twice with Buffer A (0.1% TFA) and then eluted with Buffer C (50% acetonitrile, ACN, and 0.1% TFA). Desalted peptides were lyophilized with a CentriVap Centrifugal Vacuum Concentrator (Labconco) overnight. Purified recombinant 3R and 4R tau was used as positive controls to confirm peptide identification via unique tandem mass spectrometry (MS/MS) profiles. Recombinant 2N4R tau was purchased from SignalChem, and it was maintained in the manufacturer storage buffer (50 mM Tris‐HCl, pH 7.5, 150 mM NaCl, 0.25 mM DTT, 0.1 mM phenylmethylsulfonyl fluoride [PMSF], 25% glycerol). 1N3R tau was purchased from rPeptide and resuspended in 100 mM Tris‐HCl pH 8.0 buffer (Invitrogen). Five micrograms of total recombinant protein and 0.1 µg of enzyme were used per sample for digestion.

Each sample was analyzed on a Q‐Exactive HFX mass spectrometer (Thermo Fisher Scientific) fitted with a Nanospray Flex ion source and coupled to an M‐Class Acquity liquid chromatography system (Waters Corporation) essentially as described.^48^, ^49^ The peptides were resuspended in 40 µL of loading buffer (0.1% TFA), and 1 µL was loaded onto a Waters CSH 1.7 µm C18 column (150 µm x 15 cm). Elution was performed over a 10‐min gradient at a nominal rate of 1500 nL/min, with Buffer B ranging from 1% to 20% (Buffer A: 0.1 FA in water; Buffer B: 0.1% FA in ACN) followed by a 5 min 99% Buffer B wash. The mass spectrometer was set to collect in parallel reaction monitoring (PRM) mode with an inclusion list consisting of each peptide (Table S2). An additional full survey scan was collected to assess for possible interference. Full scans were collected at a resolution of 15,000 at 200 mass to charge (m/z) with an automatic gain control (AGC) setting of 1 × 10^5^ ions and a max ion transfer (IT) time of 22 ms. For PRM scans, the settings were: resolution of 30,000 at 200 m/z, AGC target of 1 × 10^6^ ions, max injection time of 64 ms, loop count of 4, MSX count of 1, isolation width of 1.6 m/z, and isolation offset of 0.0 m/z. A pre‐optimized normalized collision energy of 28% was used to obtain the maximal recovery of target product ions.

Peptide eluents that contained heavy peptide pools were separated on a custom‐made fused silica column (10 cm × 150 µM internal diameter [ID] packed with Dr Maisch 1.5um C18 resin) by a Vanquish Neo (Thermo Fisher Scientific). Elution was performed over a 4 min gradient. The gradient was from 3% to 20% solvent B. Peptides were monitored on an Orbitrap Astral mass spectrometer (Thermo Fisher Scientific). Each cycle consisted of one full scan (MS1) and was performed with an m/z range of 300–750 at 240,000 resolution at standard settings and Astral HCD tandem scans for all peptide and standard targets. The higher energy collision‐induced dissociation (HCD) tandem scans were collected at 30% collision energy with an isolation of 1 m/z, a resolution of 80,000, an AGC setting of 1000% normalized AGC target, and a maximum injection time set to 10 ms. PRM targets were scheduled with 1 min windows (± 30 s) with an inclusion list containing both heavy and light precursors (Table S3).

### 2.8 Immunofluorescence (IF)

Slides used for staining contained 4  µm coronal sections of paraffin‐embedded tissue from the marmoset brain. Sections were deparaffinized (100% Xylene; 2 × 5  min) and dehydrated in serial ethanol dilutions (100%, 95%, 70%, 50%, and double distilled water (ddH2O) for 3 min each). Sections were incubated in boiling antigen retrieval buffer (10 mM sodium citrate, 0.05% Tween 20, pH 6.0) for 30 min in a water bath at 95°C. Slides were then blocked (10% Normal Goat Serum in 1x PBS (Phosphate‐buffered saline) for 30 min at room temperature. Sections were then incubated sequentially with each primary antibody (overnight at 4°C), and Alexa Fluor conjugated secondary antibody (1 h at room temperature on the following day). 4R tau (Cell Signaling, cat #79327) and RD3 (EMD Millipore, cat #05‐803) were diluted at 1:200 in blocking solution. Alexa Fluor conjugated 488 and 555 secondary antibodies (Thermo Fisher) were diluted at 1:1000 dilution in blocking solution. Following each incubation, slides were washed 3 × 5  min in 1XPBS at room temperature. DAPI (Fisher Scientific; 1:1000 dilution) was used for nuclei counterstaining. Slides were then incubated for 5  min in a Sudan black–based autofluorescence eliminator reagent (Millipore Sigma) at room temperature, washed 3 × 1 min in 70% ethanol, mounted (ProLong Diamond Antifade mounting media; Fisher Scientific), and stored in the dark until confocal imaging was performed. Images were obtained with a Nikon A1R HD25 confocal microscope. Acquisition settings (laser intensity, gain, and offset) were kept constant for all images within a staining group. Large, tiled images were obtained with a 20x dry objective, 1024 × 1024 scan size, resonant scanner, 8x line averaging, and 2.0 AU pinhole size. Region‐specific images were obtained with a 40x dry objective, 1024 × 1024 scan size, resonant scanner, 8x line averaging, 1.2 AU pinhole size, and 1.25x zoom. Z‐stacks were obtained with a 0.3 µm step size and images were flattened to a 2D view using maximum intensity projections (MaxIP) for representation.

### 2.9 Crude synaptosome fractionation

Crude synaptosome was isolated by sub‐cellular fractionation as described.^50^ Briefly, frozen marmoset prefrontal cortex and mouse brain tissues were homogenized using a Potter‐Elvehjem tissue homogenizer in nine volumes (weight/volume, w/v) of HEPES homogenization lysis buffer: 4 mM HEPES, pH 7.4, 2 mM EGTA, 0.32 M sucrose, 2 mM DTT with 1x Halt protease inhibitor cocktail (Thermo Fisher Scientific #78430), and 1x phosphatase inhibitor cocktail (Abcam, Waltham, MA #ab201112). The lysates were centrifuged at 1000 × *g* for 5 min at 4°C to remove nuclear material and cell debris. The supernatant (S1) was centrifuged at 12,000 × *g* for 15 min at 4°C, yielding supernatant (S2) and Pellets (P2). S2 was further centrifuged at 50,000 × *g* for 30 min at 4°C, yielding supernatant (S3), a cytosolic fraction. The P2, which is the crude synaptosome fraction, was resuspended in the HEPES lysis buffer with the addition of 0.1% Triton X‐100, and rotated for 1 h at 4°C followed by 60 min centrifugation at 16,000 × *g* at 4°C, which yielded supernatant (S4) as the extra‐synaptic fraction and pellets (P4) as the postsynaptic density fraction (PSD). P4 was resuspended in HEPES lysis buffer with an additional 0.3% Triton X‐d100. The fractions (S3, S4, P4) were analyzed by western blotting.

### 2.10 Data analysis

#### 2.10.1 RT‐PCR and WB

The DNA bands were quantified using ImageJ (National Institutes of Health [NIH]). The relative ratio of *MAPT* with and without exon 10 isoforms was derived from each band by dividing the optical density (OD) of interest from the same column. WB images were analyzed by ImageJ.

#### 2.10.2 Mass spectrometry

##### Spectral library generation

Data‐dependent acquisition (DDA) LC‐MS/MS for rTau samples were generated on an HFX Orbitrap essentially as described^49^ and imported into Proteome Discoverer (PD; Thermo, version 2.5), using the basic consensus workflow and basic QE processing workflow with the addition of SequestHT and Percolator nodes. A background proteome database of 451 proteins and all tau isoforms as incorporated for false discovery rate (FDR) correction. Only the input files and enzyme selection were adjusted between the Trypsin and LysargiNase library generations. Parameters were set to 2 maximum missed cleavages, 20 parts per million (ppm) precursor mass tolerance, and a 0.05 Da fragment mass tolerance. Variable modifications included methionine oxidation (+15.995 Da) and dynamic protein terminus modifications (N‐term acetylation +42.011, met‐loss –131.040, Met‐loss+Acetyl –89.030). Carbamidomethyl +57.021 Da on cysteines was selected as a static modification. Percolator settings relied on concatenated validation based on q‐value with a target FDR of 0.01 (strict) to 0.05 (relaxed).

##### Skyline product ion and peak selection

The subsequent PD results files were imported into Skyline (MacCoss Lab, version 23.1.0.268) for downstream analysis. Skyline settings were as follows: either promiscuous LysN or trypsin enzyme, Human 2019 background proteome, and a minimum peptide length of 6. The peptide modifications were selected to match the PD parameters. The transition settings included the selection of the first to last product ions with precursor charges of 2 and 3; ion charges of 1 and 2; and ion types of y and b. The settings also included a 5 m/z precursor exclusion window and library match tolerance within 0.1 m/z. The most intense ions from the library spectrum were selected. The SSRCalc 3.0 (300A) hydrophobicity retention time calculator was used for all peptides, and the peptide peak selection was limited to a ± 30 s time window of this predicted elution time (Table S2) and within ± 10 ppm of the library spectrum to ensure confidence in the resulting product ion peaks. Peaks were inspected manually for discordant matches. A dot product (dotp) value was used to describe the similarity of the experimental spectra to the comparative reference recombinant protein spectra, with a dotp value of 1.0 denoting the highest similarity.

Peak area report and dotp values for each sample are included (Table S4). All additional PRM raw data files, including library and Skyline analysis files, are available on synapse.org (syn52356795 and syn52895027).

Thermo RAW files from samples containing heavy peptide pools were imported directly into Skyline and identified by N‐terminal K/R residues for LysArg peptides or C‐terminal K/R residues for Tryptic peptides. Light peptide peak identities were confirmed by overlapping retention times to heavy standards. Light peptide peak areas were normalized across samples by heavy peptide relative abundance. Normalized peak areas for peptides of interest were used for quantitative proteomic analyses, including isoform ratios (Table S5). The inclusions of heavy standard peptides also illustrate a ratio dotp (rdotp) to match light product ions to heavy peptide spectra, with a perfect match being 1.0. Relative quantitation of peptide ratios and correlation across digestion approaches analysis was conducted with GraphPad Prism software (v10.1.1).

##### Base peak filtering and resulting chromatograms

Recombinant rTau RAW files were loaded into the XCalibur Qual Browser application (Thermo Xcalibur version 4.2.47, January 24, 2019). The precursor m/z from Skyline was selected for base peak filtering. The spectrum list with m/z and intensity values (Table S6) was exported and uploaded into the interactive peptide spectral annotator^51^ to generate chromatograms.

## 3 RESULTS

### 3.1 *MAPT* mRNA and protein expression in the marmoset brain

**FIGURE 1:**
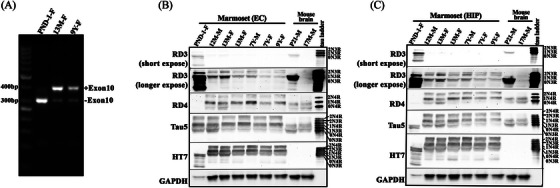
3R and 4R tau expression in marmoset brain. (A) RT‐PCR products of marmoset prefrontal cortex. Subjects are three unrelated marmosets spanning an age range of postnatal day 1 (PND1) through 9 years. The PCR products without exon 10 (304  bp) and with exon 10 MAPT (396  bp) isoforms were amplified from the cDNA library of the prefrontal cortex. Lanes are identified as follows: Lane 1, DNA ladder: PND1 (postnatal day 1, female); 13 M  (13 months old, female); 9Y (9 years of age, female). The two bands observed in each lane were indicating PCR product with or without exon10. (B) 3R and 4R tau isoform expression in marmoset entorhinal cortex (EC) and (C) hippocampus (Hip) prepared as 1% Sarkosyl soluble lysate in individual outbred wild‐type marmosets (PND1 to 9 years old), and compared to mouse brain (PND 21 and 17 months old, respectively). The PND 21 sample is a positive control for 3R tau and the 17‐month‐old sample is a negative control for 3R tau. Figure 1B and C: recombinant human tau ladder showing six tau isoforms. Row 1 is a short‐time exposure to RD3 immunoblot, and row 2 is a longer exposure to RD3 immunoblot. 3R tau was detected by anti‐3R tau‐specific antibody RD3, 4R tau was detected by anti‐4R tau‐specific antibody RD4. Total tau expression was determined by anti‐human tau‐specific antibody HT7 and anti‐tau antibody Tau5. GAPDH is a loading control. M: male; F: female.

Alternative splicing of exon 10 of *MAPT* results in 3R and 4R tau isoforms in the human brain. The inclusion or exclusion of exon 10 gives rise to 4‐repeat (4R) and 3‐repeat (3R) tau, respectively.^13^ To determine whether the 3R and 4R tau isoform splicing occurs in marmosets, total mRNA from the PFC of unrelated marmosets across different ages from neonate through 9 years was extracted and transcribed to the cDNA library. The exon 10 splicing was determined by RT‐PCR using a cDNA library as a template from each sample. As illustrated in Figure 1A, *MAPT* without exon 10 mRNA was detected predominately in neonatal marmoset brains relative to *MAPT* with exon 10 (Figure 1A, lane 1). In contrast, *MAPT* mRNA with exon 10 (Figure 1A, top band of lane 2, 3) was detected predominately in brains from the 13‐month and 9‐years of age marmosets, relative to *MAPT* without exon 10 (Figure 1A, bottom band of lane 2, 3). The sequence of each fragment was confirmed by Sanger DNA sequencing. The 304  bp fragment without exon 10 was 100% aligned with 0N3R *MAPT* mRNA of marmoset (MGenBank: MK630010.1), and the 397  bp fragment with exon 10 was 100% matches with 0N4R *MAPT* mRNA of marmoset (MGenBank: MK630009.1). By qPCR, 3R tau mRNA quantification in brain tissues of marmosets of different ages also demonstrated 3R tau mRNA expression in the neonate with an ≈10‐ to 15‐fold higher expression relative to 3R tau mRNA expression in the adolescent and the adult marmoset brains (Figure S2). To further verify 3R and 4R tau protein expression, the Sarkosyl soluble fraction from the hippocampus (Hip) and entorhinal cortex (EC) were evaluated in *n* = 7 marmosets of various ages and analyzed by WB. As presented in Figure 1B,C, in the neonatal marmoset brain, 3R tau was expressed predominantly relative to 4R tau in both the EC (Figure 1B) and Hip (Figure 1C), which was also observed as expected in postnatal mouse brain (PND 21), but not in adult mouse brain (17 months of age). In contrast, 4R tau was expressed predominantly in adolescent marmoset brain regions (Figure 1B,C, 12–13 months) and adult marmoset brain regions (Figure 1B,C, 7–9 years) relative to 3R tau expression, which was expressed as expected in both postnatal mouse brain and adult mouse brain regions. The total tau expression was detected by HT7 antibody (human tau‐specific antibody) and Tau5 antibody. Because HT7 antibody does not bind to mouse tau, whereas Tau5 binds to both marmoset and mouse tau, these results confirm the differential isoform expression pattern of tau in marmoset brain relative to mouse brain. These results also confirm that exon 10 splicing results in 3R and 4R tau isoforms in marmoset brain, with 3R tau predominantly expressed in neonatal marmoset brain and 3R/4R tau expressed in adolescent and adult marmoset brains.

### 3.2 Mass spectrometry of 3R and 4R Tau

**FIGURE 2:**
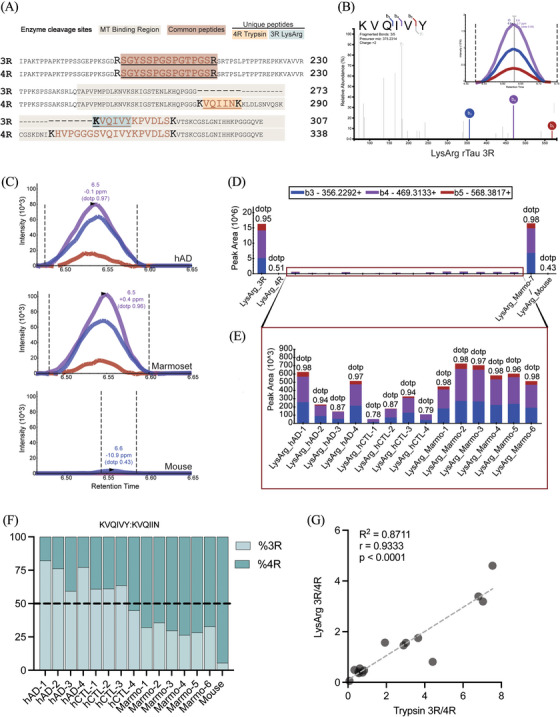
3R and 4R tau expression and ratio quantification by LysArg digestion in marmoset brain by targeted mass spectrometry. (A) Schematic diagram illustrating the primary sequence of human 3R and 4R tau. Represented is the shared 3R and 4R peptide SGYSSPGSPGTPGSR (maroon), unique 3R peptide, KVQIVY (blue), and unique 4R peptide, VQIINK (orange). (B) MS/MS spectrum of the unique 3R tau peptide, KVQIVY (m/z 375.2314, charge +2) generated by LysArg digestion of recombinant 3R tau protein. The top three product ions (b3, b4, b5) are colored. The dot‐product (dotp) value measures the similarity of the product ion pattern of the peptide to the recombinant 3R/4R tau library, with a dotp value of 1 being a perfect match. The KVQIVY product ions for recombinant 3R tau dotp value is 0.96. (C) Peak area fragment ion intensities of the KVQIVY 3R specific peptide across species. The top panel displays human AD (dotp = 0.97), the middle panel presents marmoset (dotp = 0.96), and the bottom panel shows mouse (dotp = 0.43). The human and marmoset ion fragments and peak areas are comparable to those in the recombinant 3R tau peptide. In addition, retention time matching and overlapping fragment ions confirm the co‐elution of the correct peptide in the targeted proteomics approach. (D) Overview of the unique KVQIVY 3R peptide peak area product ion intensities across samples digested with LysArg. All samples underwent retention time window filtering to ± 30s of the predicted elution time determined by the SSRCalc 3.0 hydrophobicity retention time calculator. The best peak was selected for analysis, and the strongest intensities were displayed in rTau 3R and Marmo‐7 (neonatal marmoset) samples, as expected. Recombinant 4R tau (dotp = 0.51) and mouse (dotp = 0.43) samples do not display corresponding product ions as the 3R recombinant tau. (E) Inset of peak areas plot to allow visualization of biological samples on the same scale. All marmoset and human product ions collected (b3, b4, b5) are consistent with the recombinant 3R tau (panel C). (F) 3R% (KVQIVY) and 4R% (KVQIIN) isoform abundances across adult human, marmoset, and mouse lysates. Relative quantitation was conducted through by normalizing the light peak area to respective heavy peptide. Both KVQIVY and KVQIIN peak areas were quantified from b3, b4, and b5 product ions. (G) Correlation of 3R:4R tau ratio values across LysArg and Trypsin display high consistency of ratios between digestion approaches (Pearson's *R* ^2^ = 0.87, *p* < 0.0001).

For further validation of 3R and 4R tau isoforms in the marmoset brain, a highly specific and sensitive targeted parallel reaction monitoring (PRM) mass spectrometry assay was implemented. A 3R‐specific (KVQIVY) peptide generated by LysargiNase and a 4R‐specific (VQIINK) peptide generated by trypsin, which is shared in primary sequence across mice, marmosets, and humans were selected as the targeted tau peptide sequences. A common tau tryptic peptide shared across 3R and 4R tau isoforms and species, (R)SGYSSPGSPGTPGS(R), was included to determine tau abundance across samples (Figure 2A). Of note, the KVQIVY peptide was found in the recombinant 3R tau with a near‐perfect match to the MS/MS spectrum library (dotp = 0.95) displaying b3, b4, and b5 product ions (Figure 2B). As expected, the human ion fragments and peak areas were equivalent to those in the recombinant 3R tau library with identified peaks ≤0.7 ppm and an average retention time of 6.53 min (Figure 2C, top graph). The pattern of the marmoset product ions mirrored the human results, with identified peaks less than ≤0.6 ppm and an average retention time of 6.53 (Figure 2C, middle graph). In contrast, in the mouse brain lysate, we identified a peak at –10.9 ppm, which was outside of the mass accuracy threshold (± 10 ppm) and did not correspond to the product ions of the 3R library or additional biological samples with a poor dotp of 0.43 (Figure 2C, bottom panel). As expected, neither the 4R rTau isoform nor the mouse samples matched the 3R rTau library spectrum. dotp Values were 0.51 and 0.43, respectively (Figure 2D, LysArg_4R and LysArg_Mouse). The 3R rTau isoform (LysArg_3R) (dotp = 0.95, retention time = 6.65) and neonatal marmoset (LysArg_Marmo‐7) (dotp = 0.98, RT = 6.58) displayed KVQIVY peptide peak areas of 1 × 10^6^, was 10‐ to 20‐fold higher over other marmoset and human samples. Marmo‐7 acted as a robust biological positive control for 3R tau in this study (Figure 2D). The human and marmoset samples highly matched the 3R rTau library MS/MS spectra. The average dotp values of human samples were 0.94, whereas marmoset samples were 0.98 (Figure 2E).

**FIGURE 3:**
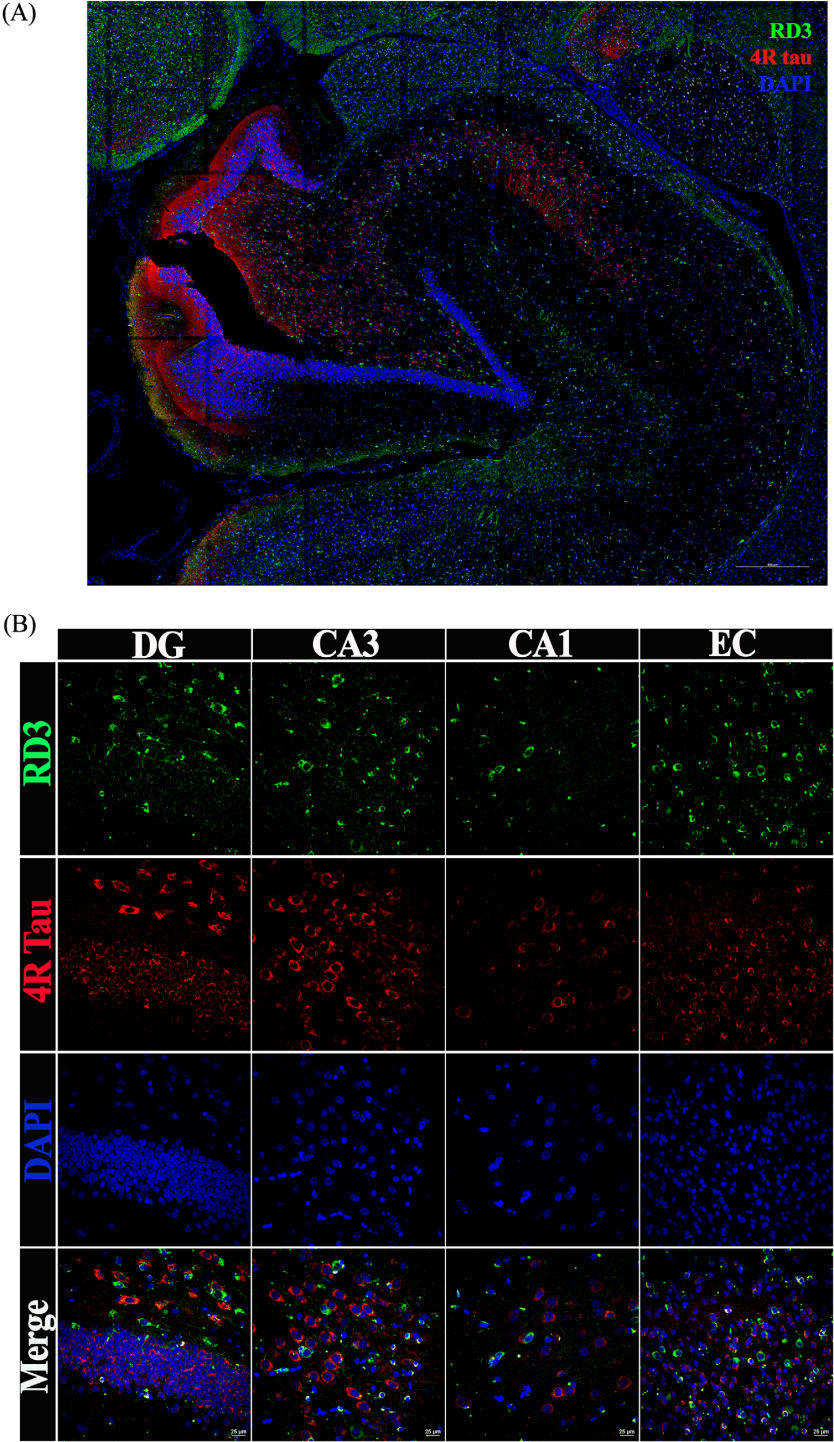
3R Tau and 4R tau expression in hippocampus and entorhinal cortex of marmoset brain. Immunofluorescence staining of 12‐year‐old male marmoset. Paraffin slides were stained with anti‐3R tau (RD3) and anti‐4R tau antibodies with Alexa488 and Alexa555 secondary antibodies, respectively. The green signals are 3R tau signals, and the red are 4R tau signals. Cell nuclei were stained with DAPI (blue). 3R and 4R tau co‐localization in the merged image (orange). The scale bar is 25 µm.

To further understand the relationship for intra‐ and inter‐species tau isoform variation, we incorporated heavy peptide standards that were isotopically labelled with either heavy lysine (K+8) or arginine (R+10), also allowing for a library‐free quantitative approach. Light peptides, generally considered to be endogenous, however, can also represent a portion of under‐labeled exogenous heavy peptide. Peak areas of light peptide were normalized to the respective heavy standard in each sample prior to visualization. Relative abundances of tau isoform ranged ≈10% across hAD cases and 12% across hCTL cases, whereas adult marmosets had 20% variation. hCTL (67.8%) and hAD (83.6%) samples overall exhibited a majority 3R tau isoform. Meanwhile adult marmosets displayed 42.6% 3R tau. Mouse lysate contained a majority 4R at 90% (Figure 2F). The average ratio dotp of light‐to‐heavy KVQIVY peptide was 0.95, and was highest in the human and marmoset samples, whereas mouse displayed a modest ratio dotp (rdotp) of 0.79 (Figure S3A). All samples matched the 4R KVQIIN heavy peptide, with a minimum rdotp at 0.83 (hCTL‐3) and mouse expressed 4R tau with a rdotp of 0.95 (Figure 3B). In addition, tau ratios were significantly correlated (R^2^ = 0.87, *p* < 0.0001) across digestion approaches (Figure 2G).

The shared 3R/4R tau peptide, SGYSSPGSPGTPGSR, was included as a tau control and displayed abundance across biological samples (Figure S4A) with abundant y7, y10, and y11 product ions (Figure S4B). The 4R‐specific peptide, VQIINK, was also included to assay the 4R tau isoform. All biological samples, mouse (dotp = 0.99), human (avg dotp = 0.98), and marmoset (avg dotp = 0.98), highly matched the 4R rTau library spectra (Figure S4C). Abundant y3, y4, and y5 product ions were represented in the recombinant MS/MS spectrum of tryptic 4R rTau (Figure S4D). The relative abundances of tau isoform from trypsin digestion had larger variation in humans, with ≈16% across hAD and 14% across hCTL cases, whereas adult marmosets had 17% range (Figure S4E). The average ratio dotp of light‐to‐heavy VQIVYKPVDLSK peptide was 0.97 and the lowest rdotp was 0.94 (Marmo‐4), whereas mouse displayed a high rdotp of 0.99 (Figure S3C). Consistent with LysArg digestion, the trypsin digested hCTL (70.7%) and hAD (82.6%) samples overall exhibited a majority 3R tau isoform. Meanwhile adult marmosets displayed a minority 3R tau (36.5%). Mouse lysate contained a majority 4R at ≈92% (Figure S4F). All samples highly matched the 4R VQIINK heavy peptide, with an average rdotp of 0.96 (Figure S3D). Taken together, these data show that humans and marmosets express both 3R and 4R tau isoforms into adulthood, with human samples under the present conditions generally displaying a higher 3R%, irrelevant of pathology.

### 3.3 Visualization of 3R and 4R tau expression in the marmoset brain

To further confirm the expression of tau isoforms in marmoset brain regions, immunofluorescent staining and immunohistochemistry were performed. As presented in Figure 3, 3R tau (green) and 4R tau (red) signals were observed in the hippocampus and entorhinal cortex regions of a 12‐year‐old male marmoset. Both 3R and 4R tau signal was observed in the soma and projections of cells across hippocampal and entorhinal cortex regions (Figure 3A). Specifically, Figure 3B merged images showcase the presence of cells that exclusively express each isoform of tau separately or an overlay of both. No 3R and 4R tau‐positive signals were observed in slices incubated without the primary antibodies and tau isoform–specific immunostaining patterns were similar using only 3R or 4R tau–specific primary antibodies (Figure S5). Immunohistochemical analysis confirmed the observation of immunofluorescent staining within the same subject (Figure S6). Notable is that the 3R and 4R tau expression patterns were similar using both methods. These data are consistent with RT‐PCR and western blot data, and MS analysis, confirming the presence of both 3R and 4R tau isoforms in the adolescent and adult marmoset brains.

### 3.4 Tau isoform expression in the synaptic region

**FIGURE 4:**
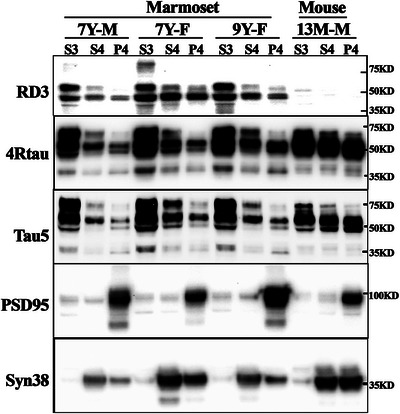
4R tau and 3R tau are expressed in the synaptic region of the marmoset prefrontal cortex. The prefrontal cortex of *n* = 3 unrelated adult marmosets was fractionated, and 3R tau and 4R tau expression was determined by RD3 and 4R tau antibodies. Adult C57BL/6J mouse brain was used as negative control for 3R tau. S3: cytosolic fraction; S4: Extra‐synaptic fraction, determined by an anti‐synaptophysin antibody; P4: postsynaptic density fraction, determined by the anti‐PSD95 antibody. Total tau was determined by the anti‐Tau5 antibody. 7Y‐M, 7‐year aged male marmoset; 7Y‐F, 7‐year aged female marmoset; 9Y‐F, 9‐year aged female marmoset; 13 M‐M, 13‐month aged male C57BL/6J mouse.

Increasing evidence indicates that tau localizes in synaptic subcellular regions and plays essential roles in synaptic function.^29^, ^30^, ^31^, ^32^, ^33^ To identify whether 3R tau and 4R tau were localized in synaptic subregions of adult marmoset neurons, a crude synaptosome was extracted from the PFC, then further fractionated to extra‐synaptic and post‐synaptic density fractions, and analyzed by WB. As illustrated in Figure 4, 3R and 4R tau was observed in the synaptic region (fraction S4, P4) from three unrelated adult marmosets (ages 7 to 9 years). 3R tau was primarily expressed in the cytosolic fraction, and also distributed in the synaptic region, albeit with individual variation. As expected, no 3R tau isoform was detected in mouse brain extracts (Figure 4). These data confirm the expression of both 3R and 4R tau isoforms in synaptic regions of the marmoset brain.

### 3.5 Tau phosphorylation and oligomerization in the marmoset brain

**FIGURE 5:**
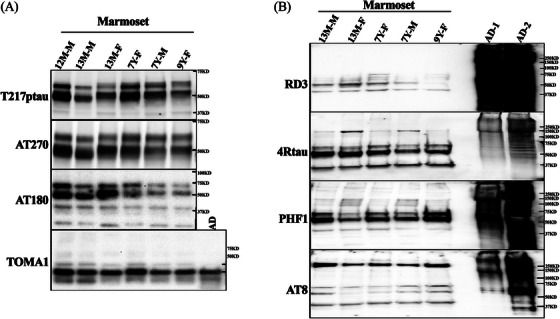
Phosphorylated tau in Sarkosyl soluble and insoluble fractions from marmoset brain. Adolescent and adult marmoset hippocampus (Hip) was extracted as 1% Sarkosyl soluble and entorhinal cortex (EC) insoluble fractions in individual outbred unrelated marmosets and analyzed via western blots. (A) The soluble extract of *n* = 6 marmosets. 12 M‐M, 12‐month aged male; 13 M‐M, 13‐month aged male; 13 M‐F, 13‐month aged female; 7Y‐F, 7‐year aged female; 7Y‐M, 7‐year aged male; 9Y‐F, 9‐year aged female. The phosphorylation sites were determined by AT270, an anti‐Thr‐181 phospho‐tau monoclonal antibody; Thr‐217 pTau, an anti‐Thr‐217 ‐pTau antibody; and AT180: anti‐Thr‐231 pTau antibody. The tau oligomer in the soluble fraction was confirmed by antibody TOMA1, an anti‐human oligomeric tau monoclonal antibody. (B) Hyperphosphorylated high‐molecular‐weight tau aggregates were detected in Sarkosyl insoluble fraction of adult marmoset entorhinal cortex by AD tau‐specific antibody PHF1 and AT8. RD3 and anti‐4R tau antibodies confirmed the presence of 3R and 4R tau in the tau aggregates. Lanes 1 to 5 are marmoset samples from different ages and sexes: 13 M‐M, 13‐month aged male; 13 M‐F, 13‐month aged female; 7Y‐F, 7‐year aged female; 7Y‐M, 7‐year aged male; 9Y‐F, 9‐year aged female; Lane 6 (blank); Lane 7‐8, two AD patient frontal cortex extraction (AD1 and AD2).

Phosphorylation of tau has been associated with tau misfolding, accumulation, and formation of AD‐like pathology.^52^, ^53^, ^54^ To evaluate whether similar tau phosphorylation sites are present in the marmoset brain, the Sarkosyl soluble fractions were analyzed by WB. As presented in Figure 5, tau phosphorylation sites (Thr181, Thr231, Thr217) were phosphorylated in the soluble fraction of adolescent and adult marmoset brain (Figure 5A) and present with oligomer‐like properties, as detected by the tau oligomer‐specific monoclonal antibody TOMA1. Similar properties were observed in AD brain extract (Figure 5A). To identify the possibility of the formation of high molecular weight tau aggregates, the Sarkosyl insoluble fractions were analyzed by WB, with PHF1 and AT8 antibodies (Figure 5B). The 1% Sarkosyl insoluble tau in entorhinal cortex extracts from *n* = 5 adolescent and adult marmosets were phosphorylated at Ser‐396/Ser‐404, and presented with a diversity of high‐molecular‐weight aggregates in all marmoset brains (Figure 5B, top panel). A 250 kD (Kilodalton) high‐molecular‐weight band for AT8 positive tau was detected in all marmoset brains (Figure 5B). To confirm the 3R and 4R tau isoform expression in Sarkosyl insoluble fraction, anti‐3R tau and anti‐4R tau antibodies were used. Smeared 4R tau bands were observed in all samples, including human AD brain extracts, and intense bands at 37 kD, 55 kD, and 60 kD were observed in the brains from the adolescent (13 months) and the adult (7–9 years) marmosets. Robust smeared RD3 signal was observed in human samples, with only low‐molecular‐weight bands (40–70 kD) observed in the marmoset samples (Figure 5B). These data indicated that Thr181, Thr231, Thr217, Ser202/Thr205, and Ser396/Ser404 phosphorylation sites were phosphorylated in the marmoset brain, with evidence of the formation of high‐molecular‐weight aggregates.

## 4 DISCUSSION

The present study is the first to confirm the expression of both 3R and 4R tau isoforms in the marmoset brain across a range of the ages throughout the marmoset lifespan. These results emphasize the relevance of the marmoset as a model system for the study of AD and related dementias.^1^

Few studies have evaluated tau expression and localization in the marmoset brain, and only one study focused on tau isoform expression.^9^, ^10^, ^11^, ^12^ In contrast to that single report indicating that marmosets do not express the 3R tau isoform as adults, which was a limited analysis of only two marmosets,^10^ our comprehensive evaluation of tau isoform expression in eight unrelated individual subjects across age ranges using multiple methodologies inclusive of RT‐PCR, DNA fragment sequencing, western blot, immunohistochemistry, mass spectrometry, synaptosome fractions, all consistently verify the presence of both 3R and 4R tau isoforms in the marmoset brain.

The main difference between the previous report that failed to detect 3R tau isoform expression in adult marmoset brains and our present results may be related to the detection methods and reagents used. Specifically, in the present study, we not only observed the DNA bands from RT‐PCR products corresponding to mRNA of marmoset 3R tau and 4R tau but also amplified and sequenced their DNA fragments, which were matched with published marmoset *MAPT* mRNA isoforms from the MGenbank database (MK630010.1 and MK630009.1).^10^ Furthermore, we examined tau isoforms in the Sarkosyl soluble and insoluble fractions, which is a standard protocol used to extract pathological tau from human AD brain tissue,^38^, ^45^ and immunofluorescent staining and immunohistochemistry to visualize the 3R and 4R tau in brain tissue (Figure 1D). In addition, we performed a precise targeted MS analysis via parallel reaction monitoring to confirm and extend these observations, including identifying 3R and 4R peptide sequences that we demonstrate are conserved between marmoset and humans. It is important to note that even with these robust methods, the presence of pathological tau aggregates in these marmoset brain samples was scarce,^4^ especially relative to what is typically observed in the brains of AD patients.^55^, ^56^, ^57^ This discrepancy may very well be related to the marmoset age at the time of death in the present set of experiments, as well as their limited longevity in captivity, because other laboratories have also reported only mild presentations of pathological tau in the marmoset brain along with significant variability across individuals; which may also be related to variations in tissue preparation and fixation methods.^9^, ^11^, ^25^, ^58^ In addition to immunofluorescent staining results, we also observed AT8‐ and PHF1‐positive aggregates in the Sarkosyl insoluble fraction along with 3R and 4R tau isoform expression (Figure 5). These findings suggest the possibility of formation of AD‐like pathological tau aggregates in marmoset brain at an early stage. Relatedly, the present observation of 3R and 4R tau expression in synaptosomes provides insight into potential trans‐synaptic propagation, which is recognized widely as a consequence of tau pathology trans‐synaptic propagation and subsequent synaptic dysfunction in AD.^36^, ^59^ Ongoing studies in our laboratory using both genetic and tau seeding approaches will ultimately help to understand whether the marmoset is susceptible to tau aggregation, propagation, and significant spreading, which may otherwise be attenuated due to their short lifespan in captivity relative to other non‐human primate species, which have more extensive neurofilament light presentation.^11^, ^23^, ^24^, ^25^, ^60^, ^61^, ^62^, ^63^

Extending our observation of the presence of both 3R and 4R tau isoforms in marmoset brain, we sought to understand if marmosets recapitulate similar 3R/4R tau ratios as described in human brain that vary across healthy and pathological conditions. Alternative *MAPT* exon 10 splicing is a complex process regulated by short cis‐elements present both in exon 10 and in introns 9 and 10. In humans, 3R tau is expressed predominantly in fetal and neonatal brains, whereas 3R and 4R tau is expressed in the adult brain in roughly equal proportions in the absence of pathological tau, depending on brain region, methods and approaches, and tissue preparation and extraction protocols.^13^, ^14^, ^38^, ^64^, ^65^ Our present results are consistent with the findings of predominant 3R expression in the neonate, whereas the adolescent and adult marmoset samples had more similar proportions of 3R and 4R (of ≈40% and 60%, respectively) as measured by MS (Figure 2). This may indeed be the normative physiological expression pattern of 3R/4R tau in healthy aging marmoset PFC, although further detailed quantification will be required to confirm the transcription and translation levels of 3R and 4R tau isoforms across colonies, as well as to understand if specific pathological conditions may result in a divergent ratio. Relatedly, this may also provide an additional explanation for the lack of observation of the 3R tau isoform in adult marmosets, as reported previously.^10^ Marmoset populations are genetically diverse and typically maintained in the laboratory as outbred colonies. Given that genetics may play a role in tau isoform expression, at least in humans, it is possible that whole genome sequencing (WGS) data can also reveal insights into the differences across studies. However, WGS data have not yet been reported.

Hyperphosphorylation of tau is associated with functional changes related to pathological conditions.^30^, ^52^, ^53^, ^54^, ^55^, ^56^, ^57^, ^58^, ^59^, ^66^, ^67^, ^68^ Phosphorylation in several residues contributes to the formation of pathological tau aggregates in the human AD brain, notably at Thr217, Thr181, and Thr231, which have also been demonstrated as biomarkers of AD in tissues and fluids.^25^, ^41^, ^42^, ^68^ In the present study, we demonstrate that these phosphorylation sites are naturally phosphorylated in the hippocampus of adolescent and adult marmosets. These results confirm and extend previous immunohistochemistry reports of hyperphosphorylated Thr231 tau in adolescent and aged marmoset brains.^9^, ^10^, ^11^ Furthermore, we also confirmed with AT8 and PHF1 epitopes the phosphorylation in Sarkosyl‐insoluble fractions in adolescent and adult marmoset entorhinal cortex, which reproduces previous findings of AT8 expression in marmoset brain by immunohistochemistry.^11^

As described above, there were a number of limitations to the present studies. First, the tissues used from these studies were not from planned terminal experiments of the subjects, but provided from the National Institute on Aging (NIA)–funded MARMO‐AD consortium tissue biobank including the tissue of the deceased neonate.^5^ Relatedly, the sample obtained from the neonate was recovered after death with a longer post‐mortem interval than the other samples as described in the Methods section and was therefore excluded from specific analyses. Despite this, the higher expression of 3R over 4R in the neonate is in line with previous reports.^10^ It is important to note that although these tissues provided the appropriate samples to address the primary aims of the study, which was the verification of the presence and/or absence of 3R and 4R tau isoforms in marmoset brain, the sample size was not powered sufficiently to fully investigate age‐dependent changes on the expression of tau isoforms across the lifespan. Indeed, additional studies will be needed to further characterize tau pathology in aging marmosets including both physiological levels and under pathological conditions. Second, it is important to note that our quantification of the 3R/4R ratio was limited to the prefrontal region of the marmoset brain using an 8 M urea tissue preparation in marmosets that were devoid of pathological tau aggregation. Although MS allows for very precise quantification, it is possible that regional differences across the brain may vary in the 3R/4R ratio under both physiological and pathological conditions. Furthermore, the tissue preparation including the buffers used and selection of fraction (e.g., soluble vs insoluble vs tissue lysate) may contribute to divergent quantification across studies including the predominance in the 3R isoform under non‐detergent conditions of the human control and AD samples in the present experiments. We point out here that our tissue preparation was with a strong chaotropic agent, 8 M urea, without aid of detergents or fractionation, which has been used traditionally for tissue lysis prior to studying tau isoform abundances.^38^, ^45^ Irrespective of these differences, the present studies confirm through multiple methodologies that the marmoset brain expresses both 3R and 4R tau isoforms.

Although further research is necessary to thoroughly investigate the role of pathological tau formation in the marmoset and the trajectory of tau neurotoxicity that leads to neurodegenerative diseases, taken together, these findings highlight the importance of the marmoset as a model system for studying primate‐specific mechanisms of AD and tauopathies, as well as the benefit of the marmoset for evaluating interventions aimed at stopping or preventing AD.

## CONFLICT OF INTEREST STATEMENT

S.J.S.R. has served as a consultant for Hager Biosciences, GenPrex, Inc., and Sage Therapeutics and holds shares in Momentum Biosciences. G.W.C. has served as a consultant for Astex Pharmaceuticals. N.T.S. and D.D. are co‐founders and board members of Emtherapro Inc and Arc Proteomics. H.H., S.M.S., F.W., S.‐H.C., S.K., J.K., Y.M., T.R.G., C.M.W., A.T., L.K.H.S., and A.C.S. report no competing interests to declare at the time of submission. Author disclosures are available in the supporting information.

## HUMAN SUBJECT CONSENT

De‐identified tissue was provided through approved resources at the University of Pittsburgh and Emory University that are exempt

## Supporting information



Supporting Information

Supporting Information

Supporting Information

Supporting Information

Supporting Information

Supporting Information

Supporting Information

Supporting Information
