# Supplementary figures and images for: Marmosets as model systems for the study of Alzheimer's disease and related dementias: Substantiation of physiological tau 3R and 4R isoform expression and phosphorylation
Source: Alzheimers Dement. 2024 Nov 19;21(1):e14366. doi: 10.1002/alz.14366 (PMC11782843; doi:10.1002/alz.14366)

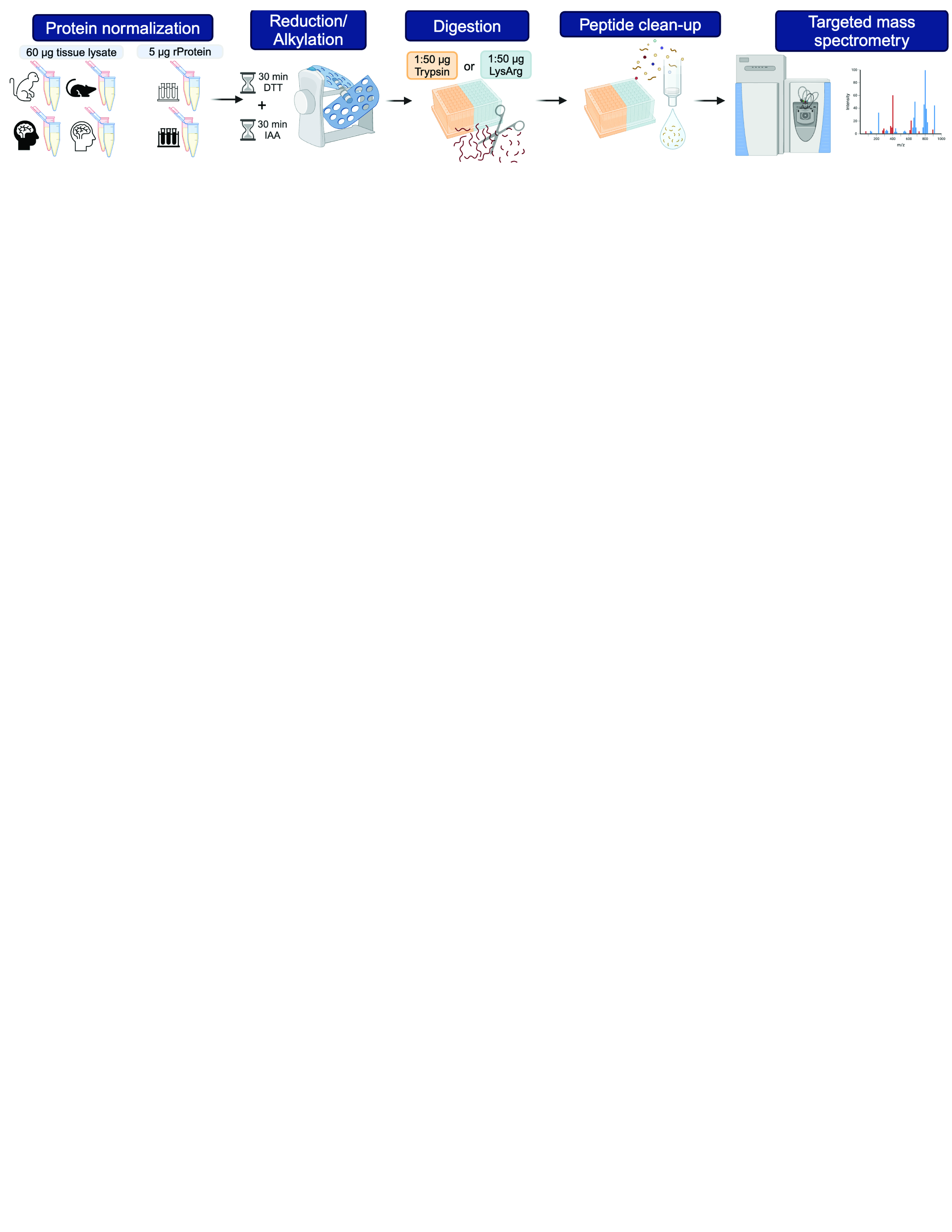

Supplement: Supplementary file 1 — Supporting Information [file ALZ-21-e14366-s001.tif]

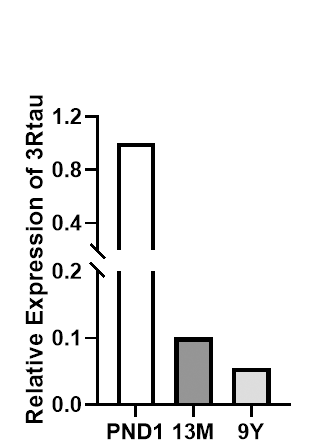

Supplement: Supplementary file 2 — Supporting Information [file ALZ-21-e14366-s008.tif]

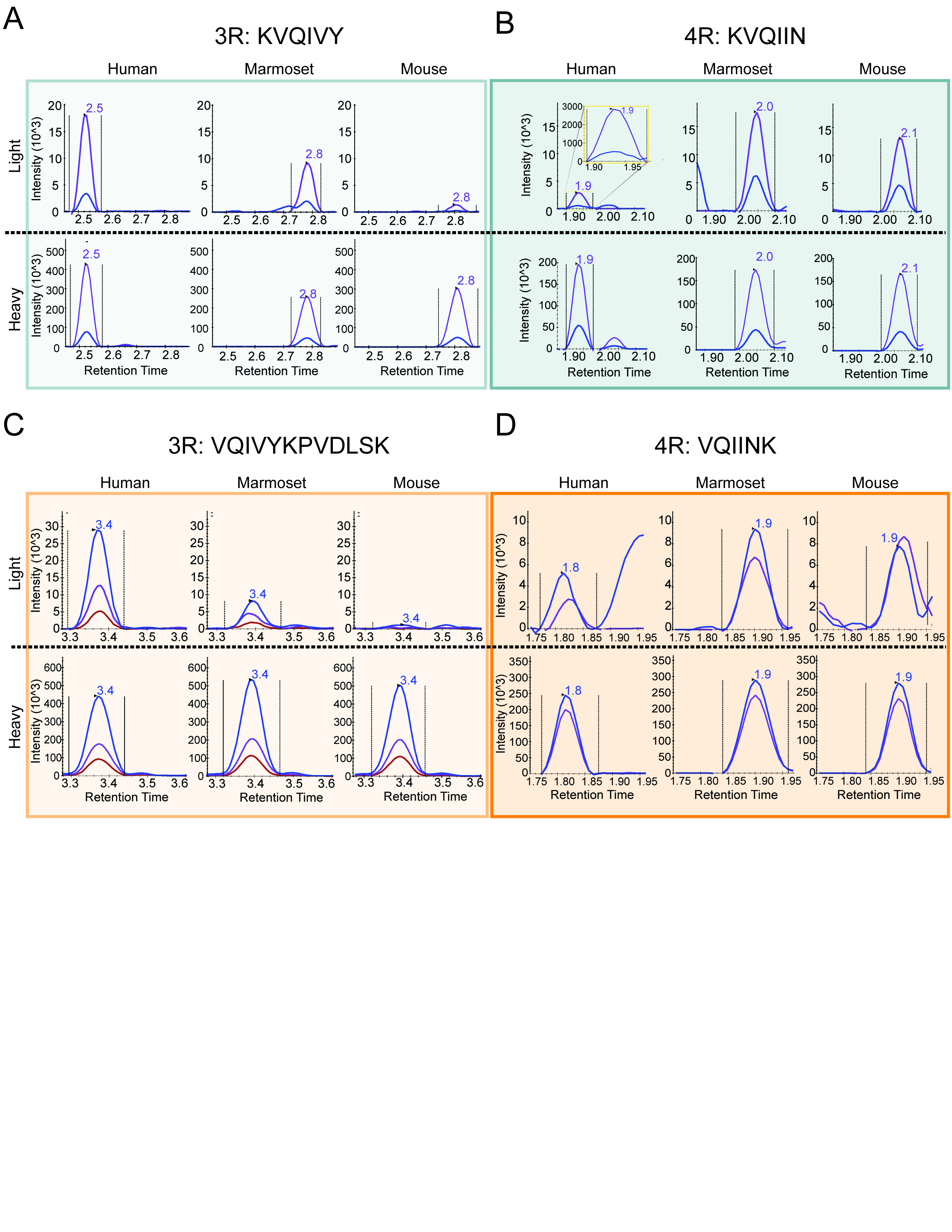

Supplement: Supplementary file 3 — Supporting Information [file ALZ-21-e14366-s003.tif]

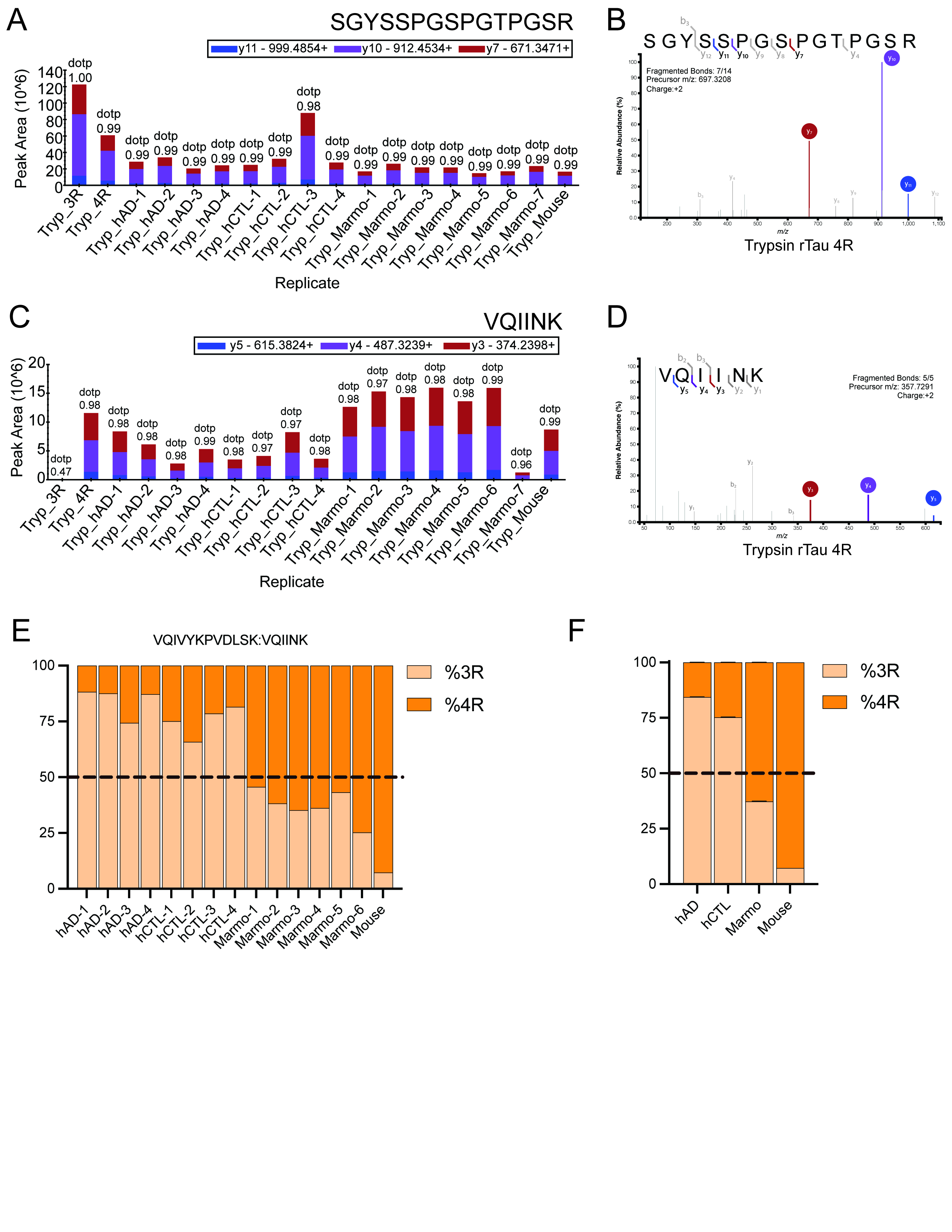

Supplement: Supplementary file 4 — Supporting Information [file ALZ-21-e14366-s002.tif]

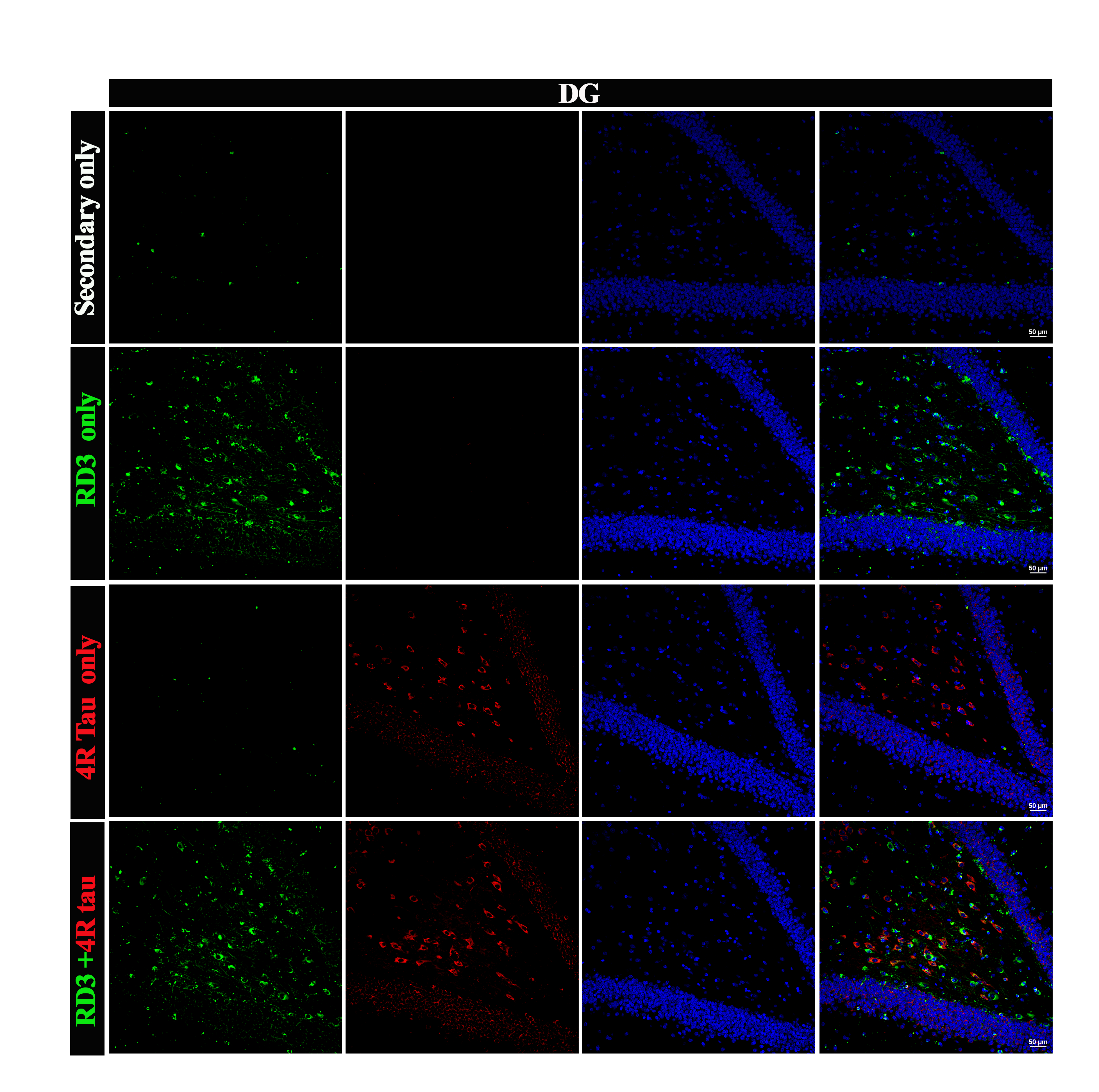

Supplement: Supplementary file 5 — Supporting Information [file ALZ-21-e14366-s006.tif]
