# Supplementary material for: Marmosets as model systems for the study of Alzheimer's disease and related dementias: Substantiation of physiological tau 3R and 4R isoform expression and phosphorylation
Source: Alzheimers Dement. 2024 Nov 19;21(1):e14366. doi: 10.1002/alz.14366 (PMC11782843; doi:10.1002/alz.14366)

**Supplemental Methods**

**1.1 qPCR quantification of MAPT mRNA levels**

Quantitative PCR (qPCR) was conducted using the SYBRgreen-based method by PowerUp™ and SYBR™ Green Master Mix, a qPCR thermocycler (Applied bioscience). Equal amounts of reverse-transcribed cDNA from the brain tissue of marmosets of the following ages were used as a template: one subject at postnatal day 1, one adolescent subject aged 13 months, and one aged adult of 9 years. For the 3R targeted reaction, the following 3R Tau-specific primers were used: Forward primer: gaaggtgcaaatagtctacaa; reverse primer: GAGACCCGATCTTCGACTGGAC. Peptidylprolyl isomerase A (PPIA) primer was used as the control to normalize the target mRNA expression levels: forward primer: TGCTGGACCCAACAGAAACGGT; reverse primer: AAAGCGCTCCATGGCCTCCA. Each reaction was carried out in triplicate in separate wells in a total volume of 10 µL, with 1 µL of reverse-transcribed cDNA template (1 ng/µL). A reagent without a cDNA template was included in each PCR reaction to ensure no contamination from the master mix. The relative quantity of each reaction was normalized to the relative amount of PPIA mRNA. For analysis, the mean of each triplicate reaction was averaged as the final quantity of the sample, and the formula for fold-change (2−ΔΔCt) was used to analyze the mRNA expression level of the targeted genes.

**1.2 Immunohistochemistry (IHC)**

Tissue preparation was described in Materials and Method 2.2. Paraffin-embedded 4µm brain sections were used for IHC and VECTASTAIN® ELITE® ABC-HRP Kit (#PK-6101, PK-610102)

was used for immunostaining. The sections were deparaffinized and rehydrated, followed by heat-induced antigen retrieval, then incubated in 0.3% H2O2 at room temperature for 15 min to block endogenous peroxidase activity. After incubation with 0.1% TritonX100 for 10 min at room temperature, the slices were blocked with blocking buffer: 2.5% horse serum at room temperature for 1hr, followed by incubation with primary antibodies diluted in blocking buffer at 4 °C overnight. After washing by TBST (TBS with 0.1% Tween20), the slices were immersed in biotinylated secondary antibodies diluted in TBST with 2.5% horse serum (Vector Laboratories, Inc., Burlingame, CA) for 45 min. Bound antibodies were labeled with avidin and biotinylated HRP (VECTASTAIN Elite ABC kit; Vector Laboratories, Inc.) and developed with 3,3‐diaminobenzidine (DAB) substrate (Vector Laboratories #SK-4100). Slices were mounted with VectaMount Permanent Mounting Medium (Vector laboratories#H-5000) and imaged by Zeiss Axiovert 200M microscope. Mouse anti 3Rtau antibody RD3 (1:200) and biotinylated anti-mouse secondary antibody were used for 3Rtau staining. Rabbit anti-4Rtau antibody (1:300) (Cell Signaling#79327) and biotinylated anti-rabbit secondary antibody were used for 4Rtau staining.

**
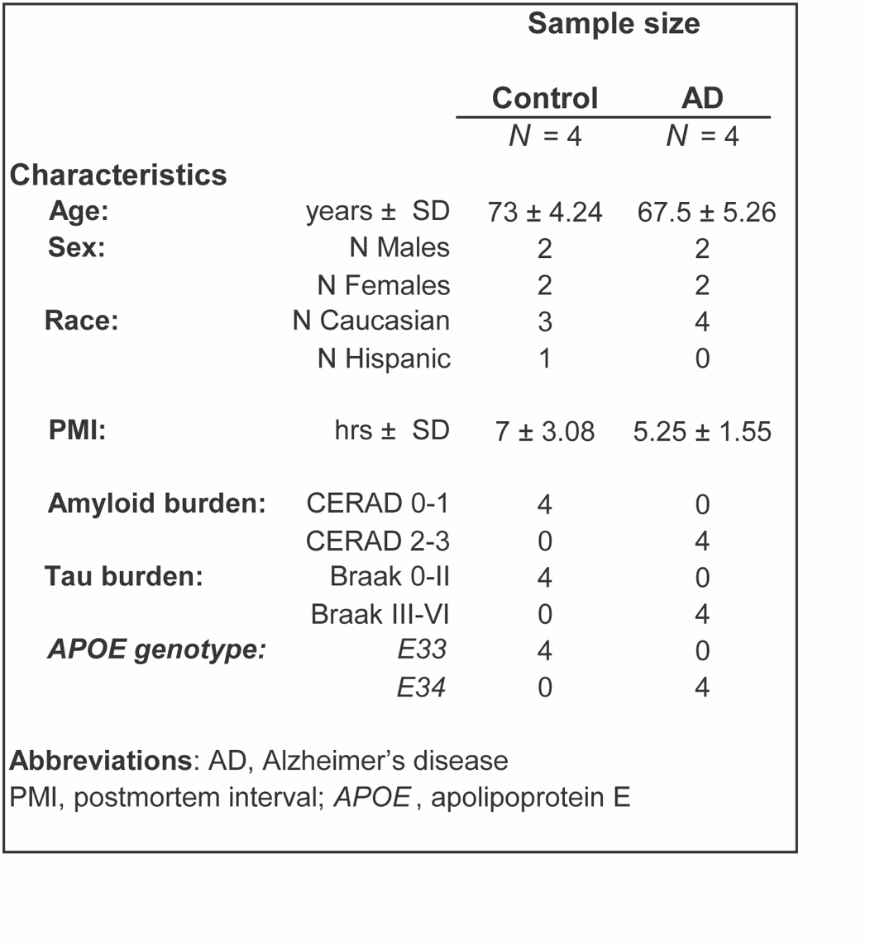
Supplementary Table 1A.** Post-mortem frozen human brain of Alzheimer’s Disease and non-demented control sections of the frontal cortex were obtained from the Emory Alzheimer’s Disease Research Center brain bank (pathological traits described in supplemental table #1B), used for Mass spectrometry and/or Western Blot.

**Supplementary Table 1B. Demographics of case traits for human tissues used (Emory cases) including primary Neuropathologic diagnosis of Alzheimer’s disease (AD) and neuropathological scoring inclusive of Braak Stage, ABC, and CERAD.** Abbreviations: PMI: postmortem interval (hours); ApoE: ApolipoproteinE status; Sex: male (m), female (f); Experimental use: mass spectrometry (MS), western blot (WB). Race: white (w), hispanic (h). NA: not applicable.


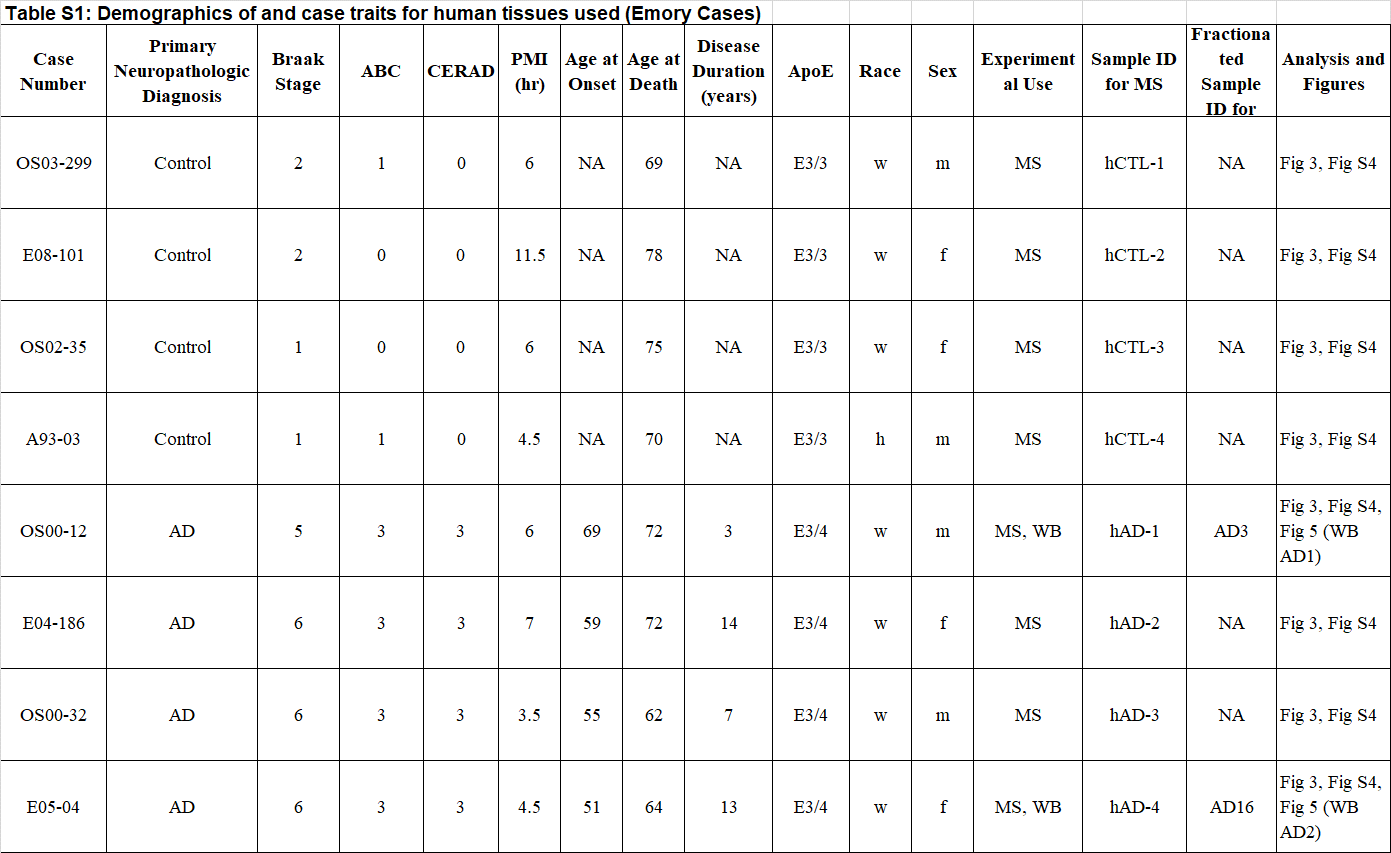


**Supplemental Figures**

**Figure S1. Schematic mass spectrometry overview using a complementary dual enzymatic digestion approach with Trypsin and LysargiNase (LysArg).** Samples (n = 18 total) including 7 Marmosets, 4 human Alzheimer's Disease (hAD) cases, 4 human non-demented control (hCTL), and 1 mouse were analyzed.


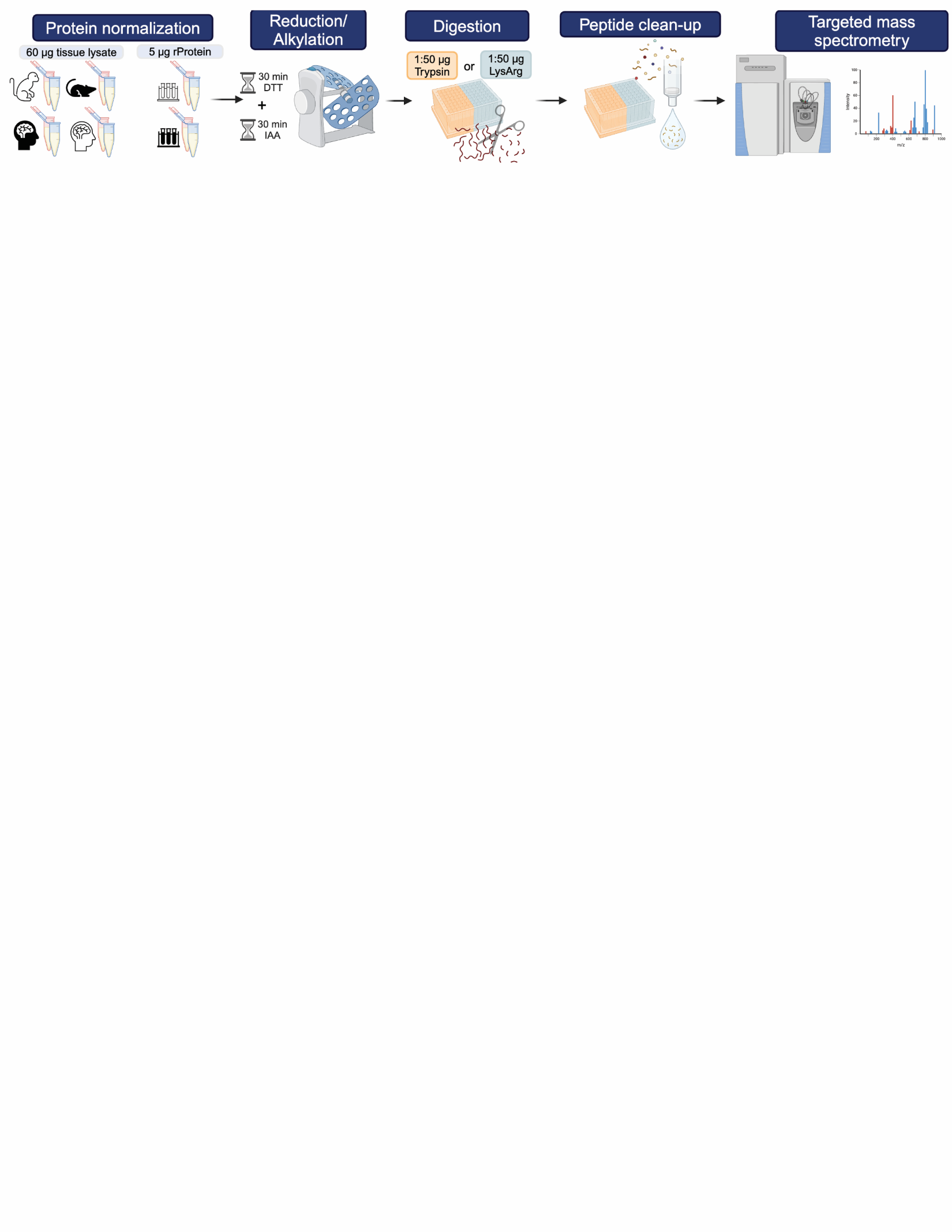


**Figure S2.** **Quantification 3R Tau expression in marmoset brain.** The mRNA expression level without exon 10 (3Rtau) was quantified by qPCR from three different marmosets spanning the following age groups: Postnatal day 1 (PND1), adolescent (13-months; 13M), and aged adult (9 years, 9Y). 3R Tau mRNA expression was 10-15-fold higher in neonate marmoset brains relative to adolescent and aged adult marmoset brain which were similar to each other.


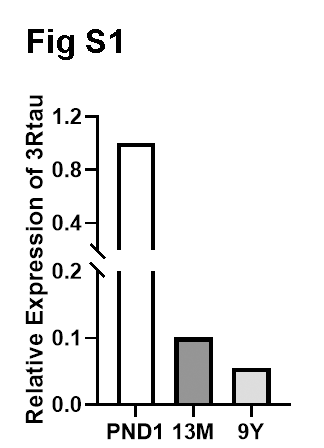


**Figure S3. Light and heavy representative peptide peaks for quantitative mass spectrometry across human, marmoset, and mouse brain lysates. A)** Representative KVQIVY peptide peaks with b4+ and b5+ product ions. The rdotp of KVQIVY peptide across all samples was 0.95, with mouse being the lowest at 0.79. **B)** Representative KVQIIN peptide peaks with b4+ and b5+ product ions. The average rdotp value for the unique 4R peptide was 0.914. **C)** Tryptic 3R VQIVYKPVDLSK peptide peaks displaying y6+, y9++, y10++ product ions. Average rdotp of 0.97. **D)** and Tryptic 4R VQIINK peptide product ions y5+, y5++, y4+, and y3+ with an average rdotp of 0.96.


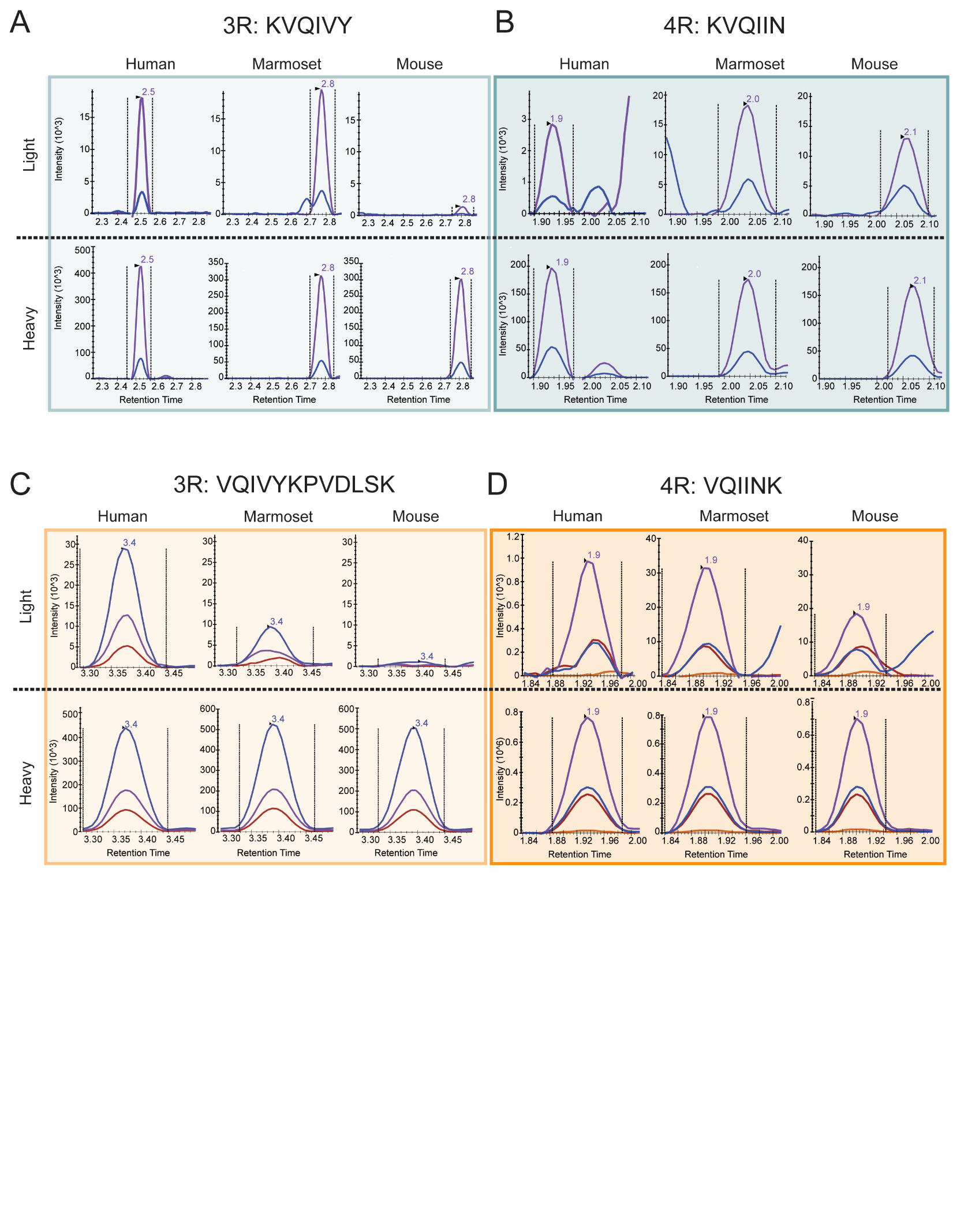


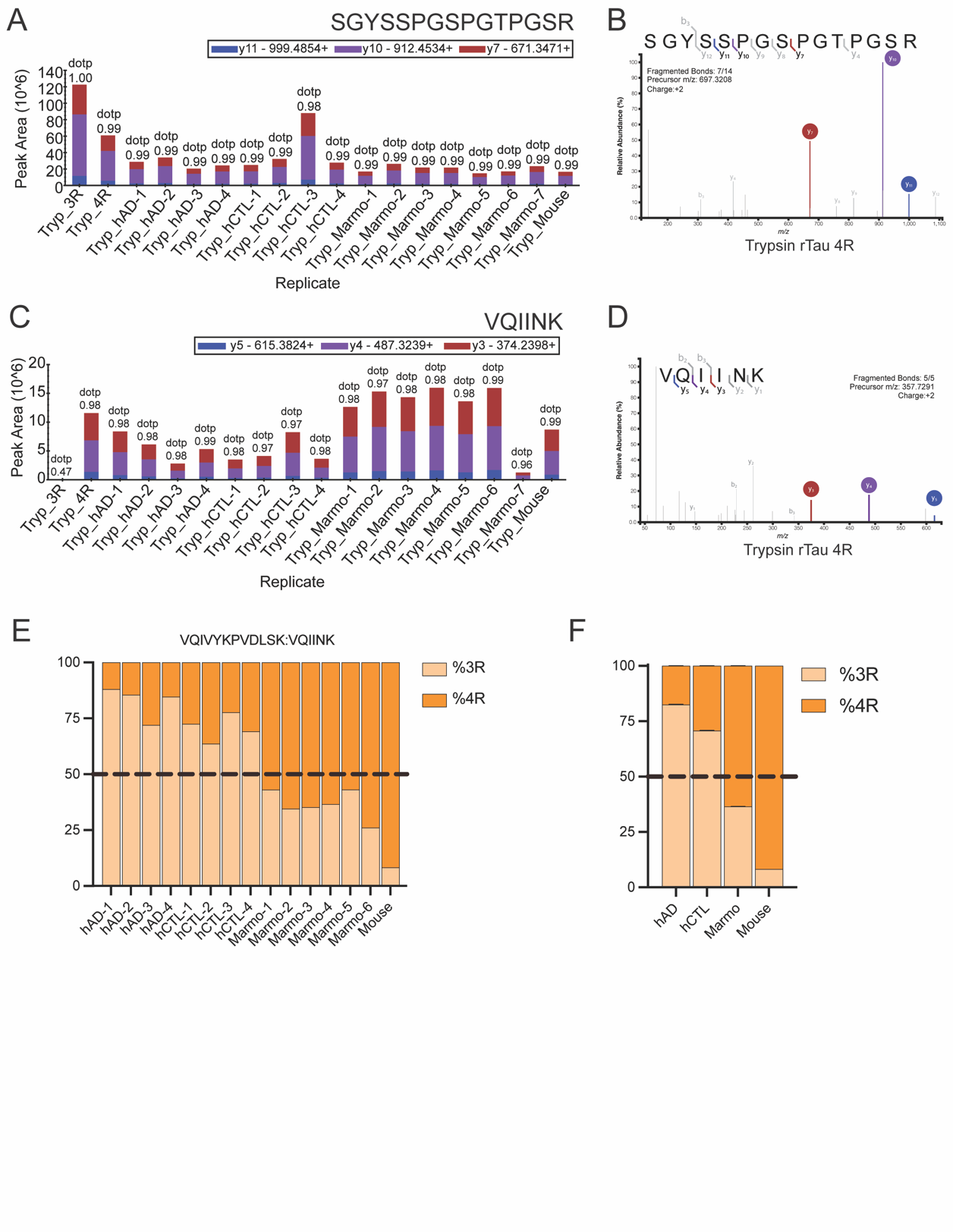
**Figure S4. 3R and 4R Tau expression by Trypsin in Marmosets by targeted mass spectrometry.** **A)** Overview of the peak area product ion intensities of the tryptic 3R and 4R shared Tau peptide, SGYSSPGSPGTPGSR, providing evidence of Tau protein abundance across all samples. **B)** MS/MS spectrum of the 3R and 4R Tau shared peptide, SGYSSPGSPGTPGSR (m/z 697.3208, charge +2) generated by tryptic digestion of 4R recombinant Tau. The top three product ions (y7(red), y10 (purple), y11(blue)) are colored. **C)** Overview of the peak area product ions of the 4R unique tryptic peptide, VQIINK. The fragment ions were consistent with the 4R Tau library across all samples except for the recombinant 3R Tau (dotp = 0.47). **D)** Representative MS/MS spectrum of Tau 4R specific, VQIINK (357.7291 m/z, charge +2) peptide generated by tryptic digestion of recombinant 4R Tau. The top three ion products (y3(red), y4(purple), y5(blue)) are colored. Samples (n = 18 total) including 7 Marmosets, 4 human Alzheimer's Disease (hAD) cases, 4 human non-demented control (hCTL), and 1 mouse were analyzed. Tryp-3R was recombinant human 3R Tau, and Tryp-4R was recombinant human 4R Tau. **E)** 3R% (VQIVYKPVDLSK) and 4R% (VQIINK) across adult human, marmoset, and mouse lysates digested with trypsin. Relative quantitation conducted through normalization of peak areas to respective heavy peptides. **F)** Averages of 3R and 4R percentages were largely unchanged in Tryptic samples. 3R% in hCTL (70.74%), hAD (82.56%), adult marmoset (36.46). Average 4R% in hCTL was 29.26, hAD was 17.44%, and marmoset was 63.54. Mouse contained a majority 4R at 91.69%.

**Figure S5. Negative control background staining of IF.** Representative confocal 20X images of background staining, in the absence of incubation with primary antibodies, for the images presented in Figure 1D-E. A 12-year aged male marmoset coronal brain section was stained with Alexa fluor 488 (Green) conjugated anti-mouse IgG and Alexa fluor 555 (Red) conjugated anti-rabbit IgG, without incubation with the primary antibodies RD3 and anti-4Rtau antibody (top panel; Secondary only). Sections were also stained with RD3 and 4R-Tau primary antibodies individually (middle panels) and with both antibodies combined (bottom panel). Images display the dentate gyrus (DG) for anatomical reference. Cell nuclei were counter stained with DAPI (blue). Scale bars are 50 µm.


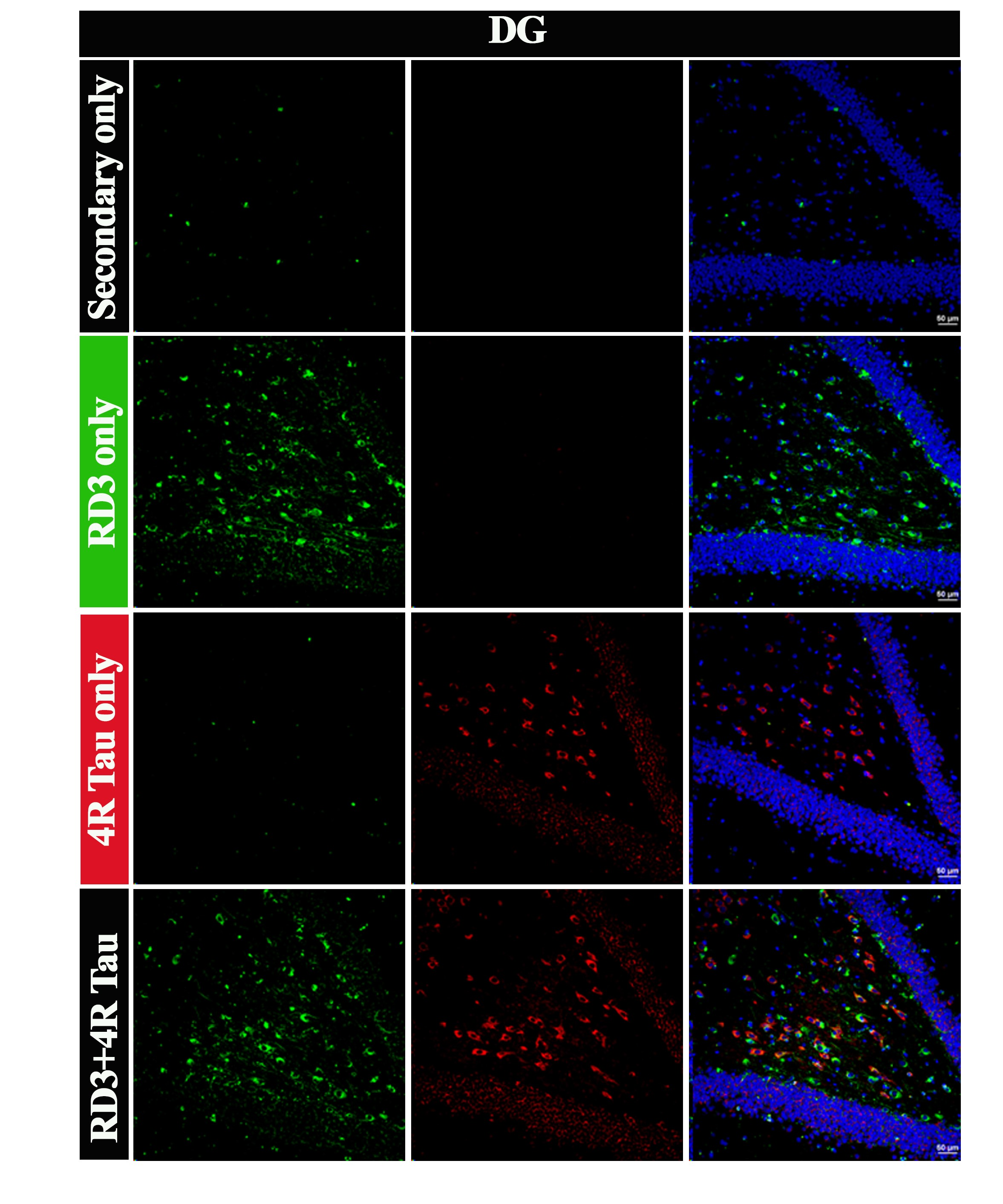


**Figure S6. Immunohistochemistry of 12-year aged marmoset brain.** As a companion to the immunofluorescence staining of the same subject as presented in Figure 2, paraffin-embedded slices from a 12-year-old male marmoset brain were stained with anti-3RTau (RD3) and anti-4RTau antibodies with ABC-HRP kit. **A and B)** 4Rtau immunostaining images in marmoset brain. The image was obtained with 2.5x objective lens, scale bar 1000 µm (**A**)**.** Representative magnified images from Hip and EC regions were obtained using a 40x objective lens, scale bar 20µm (**B**). **C and D)** 3Rtau expression detected by 3Rtau specific antibody RD3 in marmoset brain. The image was obtained with 2.5x objective lens, scale bar 1000µm(C)**.** Representative magnified images from Hip and EC regions were obtained by a 40x objective lens, scale bar 20µm (D). Cell nuclei were stained with hematoxylin (blue). CA1 is hippocampus CA1 area, CA3 is hippocampus CA3 region, DG is the hippocampus dentate gyrus region, and EC is the entorhinal cortex area.


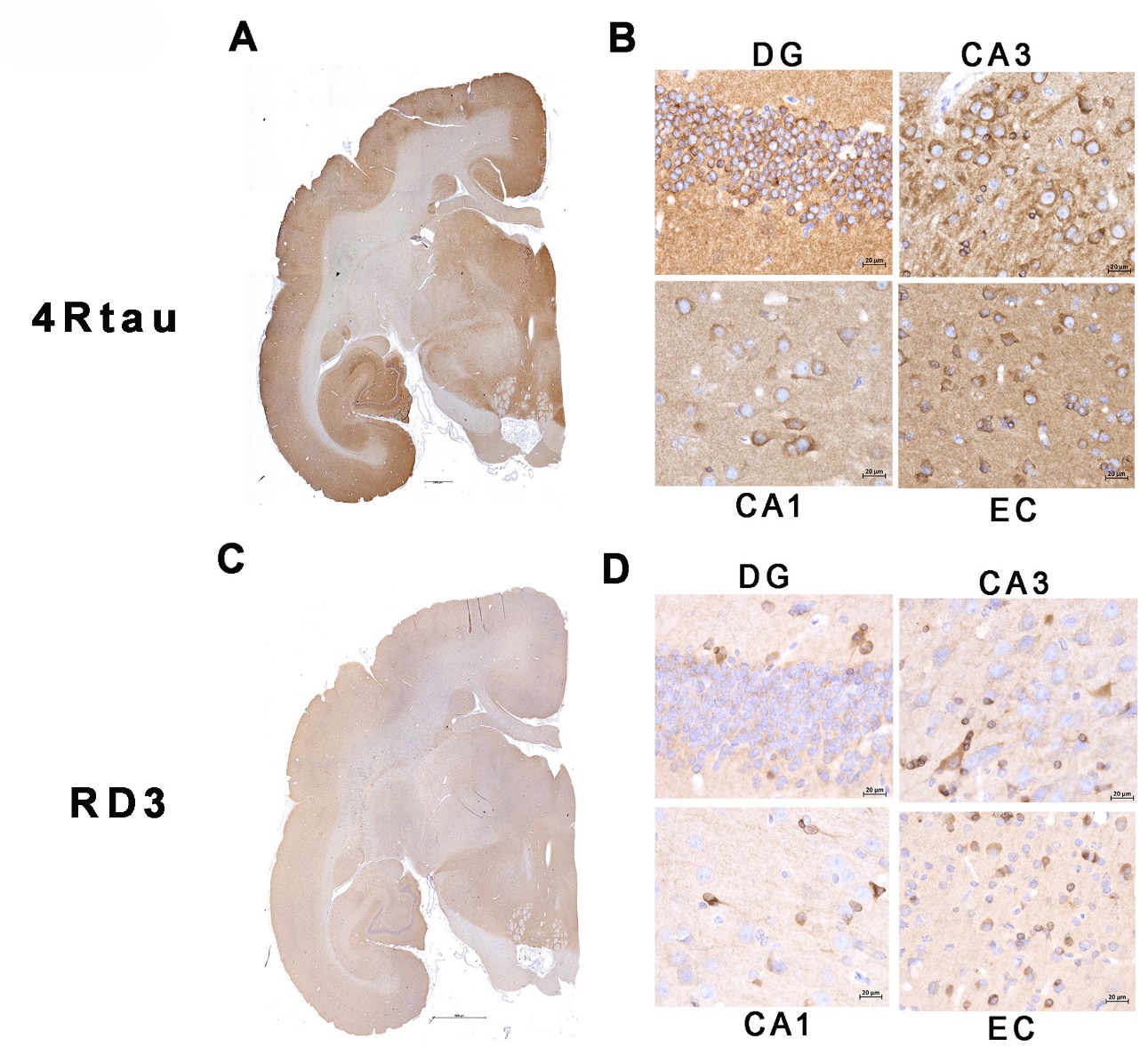

Supplement: Supplementary file 8 — Supporting Information [file ALZ-21-e14366-s007.docx]
